# Supplementary material for: An optimal growth law for RNA composition and its partial implementation through ribosomal and tRNA gene locations in bacterial genomes
Source: PLoS Genet. 2021 Nov 29;17(11):e1009939. doi: 10.1371/journal.pgen.1009939 (PMC8659690; doi:10.1371/journal.pgen.1009939)
Supplement: S1 File — (PDF) [file pgen.1009939.s001.pdf]

Supporting Information for

An optimal growth law for RNA composition and its partial implementation through ribosomal and tRNA gene locations in bacterial genomes

Xiao-Pan Hu, Martin J. Lercher

This PDF includes Figures A-K and Texts A-D.

Fig A. The effect of protein degradation on the RNA growth law in *E. coli*. 2

Fig B. Comparison of the optimal TC/ribosome expression ratio with a previously reported optimal TC/ribosome ratio for *E. coli* 3

Fig C. Gene positions of rRNA and tRNA genes in E. coli 4

Fig D. Effective population size (N_e) is not correlated with the positions of tRNA and rRNA genes. 5

Fig E. The numbers of genomically encoded rRNA genes and tRNA genes are positively correlated with μ_{max} and negatively correlated with tRNA/ribosome ratios. 6

Fig F. The tRNA/rRNA gene dosage ratio is independent of growth rate under temperature stress conditions. 7

Text A. Assessment of tRNA/rRNA gene dosage ratio calculated by a constant DNA replication rate (k_{rep} = 1000 s^{-1}) 8

Fig G. The effect of a growth rate-dependent C period on the tRNA/rRNA dosage ratio in *E. coli*. 8

Fig H. The effect of a μ_{max}-dependent DNA replication rate on the tRNA/ribosome gene dosage ratio. 9

Text B. Genome size affects gene position 10

Fig I. The relationship between genome size and the positions of tRNA and rRNA genes. 11

Text C. The positions of tRNA genes are not correlated with the corresponding codon frequencies. 12

Fig J. Copy numbers of tRNA genes but not their positions are correlated with codon frequency. 13

Text D. The effect of assuming a constant TC/ribosome expression ratio instead of an optimal ratio 14

Fig K. Cost difference (in terms of dry mass density) between the optimal TC/ribosome expression ratio and a constant TC/ribosome expression ratio in *E. coli* as a function of growth rate. 15

References 16

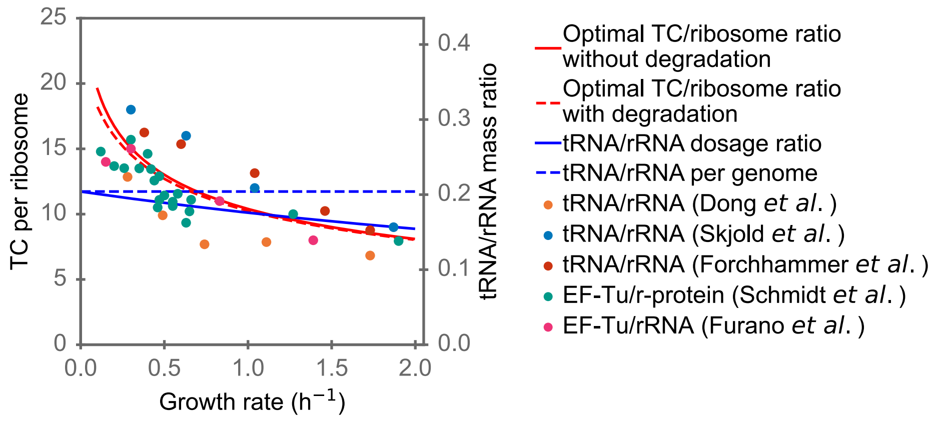

Fig A. The effect of protein degradation on the RNA growth law in *E. coli*. The solid red curve shows the RNA composition growth law as given by equation (1), which ignores protein degradation, whereas the dashed red curve shows the RNA composition growth law with protein degradation included. Protein degradation rate typically ranges from 0.02 h^{-1} to 0.04 h^{-1} [1,2] (see estimation below) and is higher at low growth rates than at high growth rates [1–3]. We here assumed a constant protein degradation rate (k_{deg} = 0.04 h^{-1}); its inclusion is equivalent to moving the optimal TC/ribosome ratio 0.04 h^{-1} to the left. As the degradation rate is relatively small compared to the maximal growth rate of *E. coli* [1,2], protein degradation affects the optimal TC/ribosome ratio only at very low growth rates, where the degradation rate becomes comparable to the growth rate.

Estimation of the protein degradation rate: Publications [1,2] report the fraction of degraded protein (f_{deg}) after time t. The protein degradation function can be written as $1 - f_{\text{deg}} = e^{-k_{\text{deg}} \cdot t}$. With this equation, k_{deg} was estimated to range from 0.005 h^{-1} to 0.04 h^{-1} according to the data reported in [2] and to range from 0.025 h^{-1} to 0.03 h^{-1} according to the data reported in [1]. To be conservative, we here chose k_{deg} = 0.04 h^{-1}, the largest reported degradation rate.

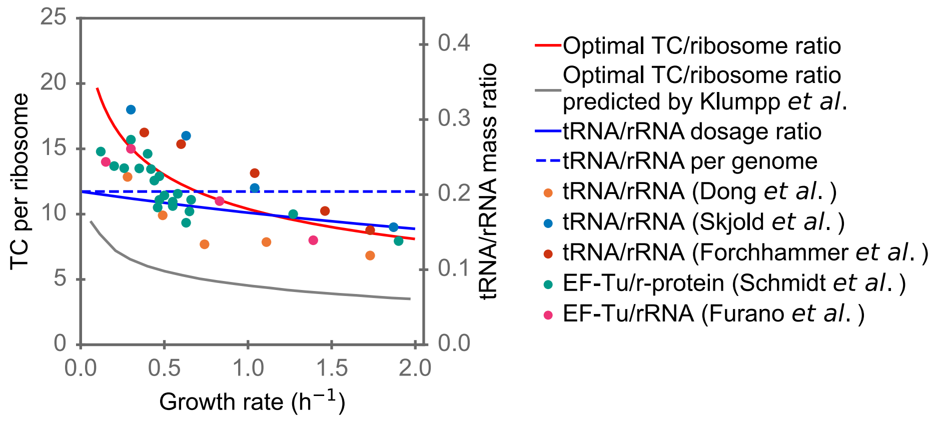

Fig B. Comparison of the optimal TC/ribosome expression ratio with a previously reported optimal TC/ribosome ratio for *E. coli* [4]. Klumpp *et. al* [4] predicted the optimal TC/ribosome expression ratio by identifying the proteome fractions of ribosome and TC that maximize growth rate in a coarse-grained, phenomenological model of cellular growth [4]. This optimal proteome allocation pattern results in a predicted TC/ribosome ratio (gray line, extracted from Fig 4C in [4]) that is substantially lower than the experimentally observed data. Here, we considered the optimal resource allocation into cellular dry mass (RNA and protein combined; red curve), motivated by the near-constant cellular dry mass density across growth conditions. The optimal dry mass allocation explains the experimentally observed TC/ribosome expression ratios much better than the optimal proteome allocation considered in Ref. [4]. Protein accounts for roughly 1/3 of the ribosome mass, whereas it accounts for roughly 2/3 of the ternary complex mass. If only the protein cost is considered, the ternary complex appears much more expensive to the cell, resulting in a lower predicted ternary-complex/ribosome ratio.

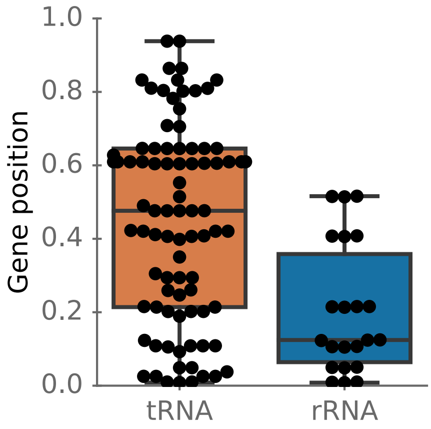

Fig C. Gene positions of rRNA and tRNA genes in E. coli. rRNA genes are located closer to oriC than tRNA genes in *E. coli*, with genomic position 0.20 ± 0.17 (mean ± standard deviation) for rRNA genes and 0.45 ± 0.27 for tRNA genes.

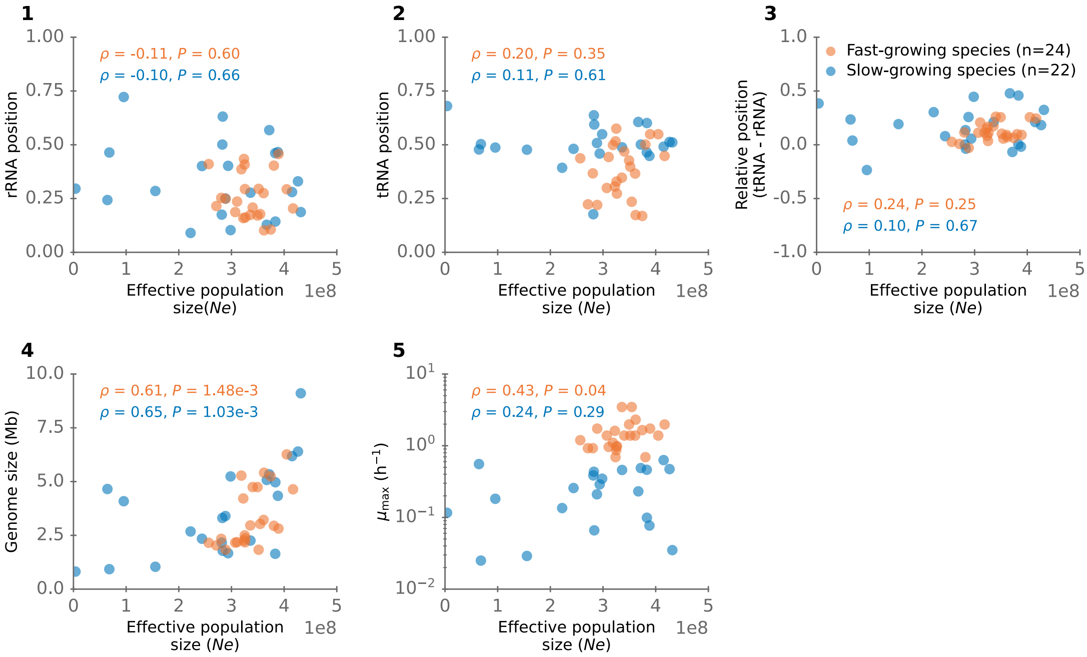

Fig D. Effective population size (N_e) is not correlated with the positions of tRNA and rRNA genes. The effective population sizes for 46 out of the 170 species were obtained from Ref. [5]. As shown in **panels 1-3**, we found no statistically significant Spearman rank correlations (P > 0.1) between N_e and the different positions considered in our study (position of rRNA genes, tRNA genes, and the relative position, $position_{tRNA} - position_{rRNA}$) when analyzing fast- and slow-growing species (P > 0.1, see colored text in panels 1-3). In contrast, we found statistically significant correlations between N_e and genome size (**panel 4**) and, for fast-growing species, between N_e and maximal growth rate μ_{max} (**panel 5**).

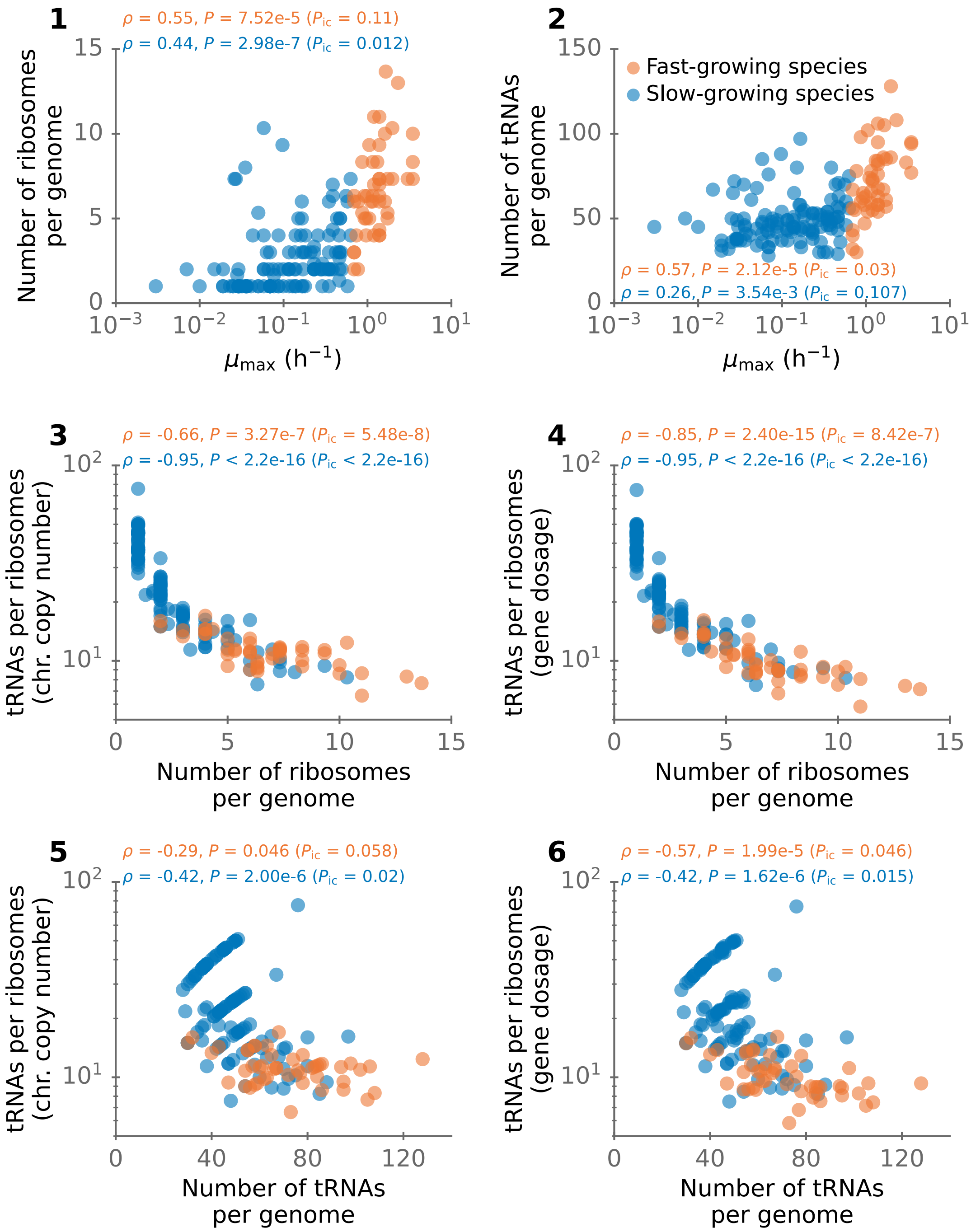

Fig E. The numbers of genomically encoded rRNA genes and tRNA genes are positively correlated with μ_{max} and negatively correlated with tRNA/ribosome ratios. With increasing maximal growth rate μ_{max}, bacterial genomes harbor more rRNA genes (**panel 1**) and tRNA genes **(panel 2). Panels 3-6** show the relationship between tRNA/ribosome dosage and genomic ratios and the number of ribosome and tRNA genes in the genome. While a negative correlation between the tRNA/ribosome ratios and the number of rRNA genes would trivially occur also if the numbers of tRNA and rRNA genes were independent (**panels 3 and 4**), a negative correlation of the tRNA/ribosome ratios with the number of tRNA genes would not be expected in this case (**panels 5 and 6**); the latter observation thus provides strong support for our hypothesis that the relative number of tRNA and rRNA genes are constrained to optimize translation efficiency.

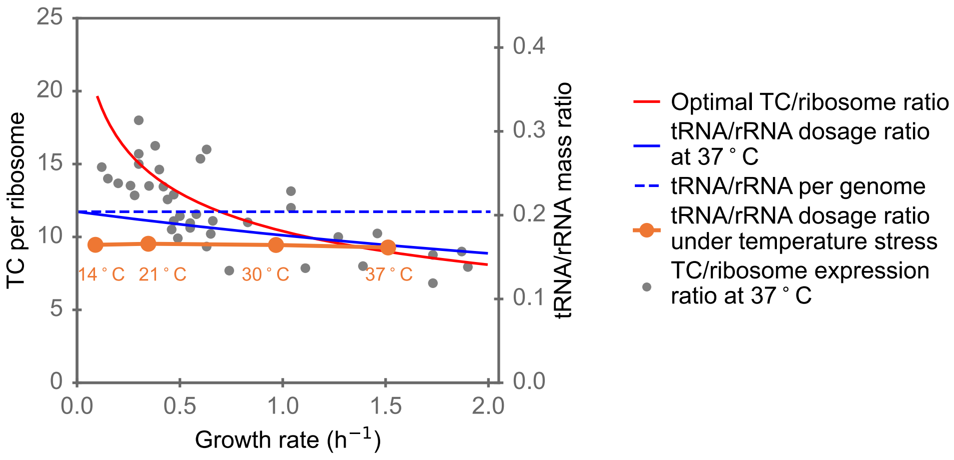

Fig F. The tRNA/rRNA gene dosage ratio is independent of growth rate under temperature stress conditions. The orange dots show experimental measurements at 14 °C, 21 °C, 30 °C, and 37 °C in the same medium [6]. The DNA replication rate became slow at low temperatures, and multiple replication rounds were observed even at low growth rates. This effect made the rRNA/tRNA dosage ratio almost independent of growth rate. As we could not find experimental data for the tRNA/ribosome expression ratio under temperature stress conditions, it is unclear if the tRNA/ribosome expression ratio is still optimal under temperature stress conditions.

Text A. Assessment of tRNA/rRNA gene dosage ratio calculated by a constant DNA replication rate (k_{rep} = 1000 s^{-1})

In the main text, we used a constant DNA replication rate (k_{rep} = 1000 s^{-1}) for all species to calculate the C period and the tRNA/rRNA gene dosage ratio (Fig 1B and Fig 3D). This is an approximation, as the DNA replication rate (1) is growth rate dependent in a given species [10,11] and (2) is species-specific and depends on the maximal growth rate (μ_{max}) across species. Here, we assess how the assumption of a constant k_{rep} = 1000 s^{-1} affects the tRNA/rRNA dosage ratio in the results shown in Fig 1B and Fig 3D.

The effect of a growth rate-dependent C period on the tRNA/rRNA dosage ratio in E. coli

In *E. coli*, the C period is almost constant at growth rates above 0.7 h^{-1} but increases with decreasing growth rate below 0.7 h^{-1} (Fig G1; data from Refs. [10,11]). However, as shown in Fig G2, when calculating the tRNA/rRNA dosage ratio using the experimentally observed rate-dependent C period, the results are very similar to those calculated under the assumption of a constant C period and DNA replication rate (k_{rep} = 1000 s^{-1}) (calculated by equation (21)). Thus, setting k_{rep} = 1000 bp s^{-1} appears to be an acceptable approximation for the tRNA/rRNA dosage ratio calculation.

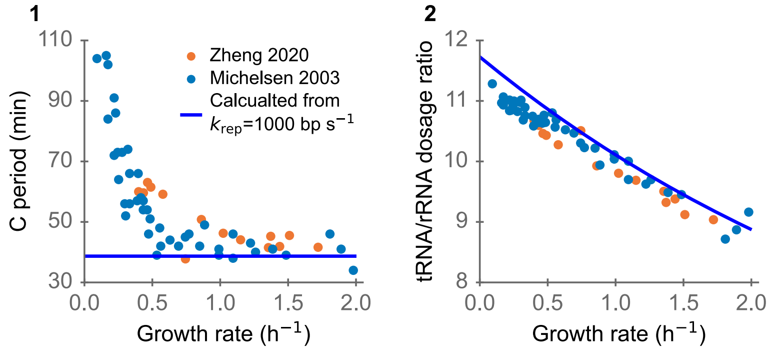

Fig G. The effect of a growth rate-dependent C period on the tRNA/rRNA dosage ratio in *E. coli*. (**panel 1**) Comparison of the C period calculated from constant k_{rep} = 1000 bp s^{-1} (solid blue line) and experimental estimates of the C period (orange and blue dots [10,11]). (**panel 2**) The growth rate-dependent C period has only a minor effect on the predicted tRNA/rRNA dosage ratio. The blue line shows the dosage ratios calculated under the assumption of a constant C period. The orange and blue dots show dosage ratios calculated using the respective experimental C period values shown in panel 1.

The effect of a species-specific replication rate on the tRNA/rRNA gene dosage ratio across species

In a literature search, we found estimates of the replication rate or C period for 5 species in our dataset, including one slow-growing species and four fast-growing species (Fig H1 and S5 Table). We fitted a linear model for the dependence of the replication rate on μ_{max} (both on log scale; Fig H1) and used this linear model to estimate the μ_{max}-dependent DNA replication rate for all species in our dataset. The tRNA/ribosome gene dosage ratio estimated using a μ_{max}-dependent replication rate (S4 Table) is very similar to the dosage estimated using a constant replication rate k_{rep} = 1000 s^{-1} (Fig H2; R^2 = 0.973). Further, the dependence of the tRNA/ribosome gene dosage ratio on the maximal growth rate μ_{max} when considering a growth rate-dependent replication rate (Fig H3, Spearman's rank correlation $\rho = -0.47,\ P = 7.0 \times 10^{-4}$ for fast-growing species; $\rho = -0.47,\ P = 5.5 \times 10^{-8}$ for slow-growing species) is very similar to that shown in Fig 3D . Thus, the dependence of the DNA replication rate on μ_{max} does not appear to affect our conclusions.

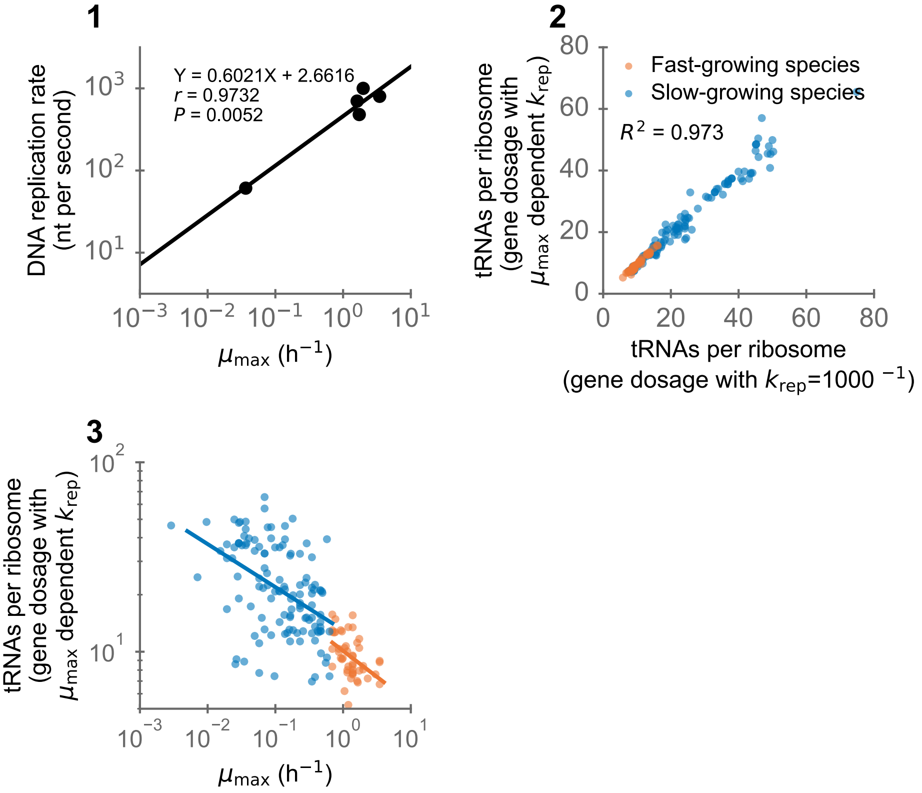

Fig H. The effect of a μ_{max}-dependent DNA replication rate on the tRNA/ribosome gene dosage ratio. (**panel 1**) linear regression on log-log scale to predict the DNA replication rate from μ_{max}. (**panel 2**) Comparison of tRNA/rRNA gene dosage ratios calculated using a constant DNA replication rate (x-axis) and using a μ_{max}-dependent replication rate. (**panel 3**) Relationship between the tRNA/rRNA gene dosage ratio calculated using a μ_{max}-dependent replication rate and μ_{max}.

Text B. Genome size affects gene position

At the same DNA replication rate, bacteria with smaller genomes need less time to replicate their DNA than bacteria with larger genomes; at the same growth rate, they hence have fewer simultaneous replication rounds in the cell. For example, *Streptococcus pneumoniae* (with a 2.1 Mb genome) does not need multiple replication rounds at its fast growth rate [7]. Thus, replication-associated gene dosage effects will be less important in bacteria with small genomes, and the positions of tRNA and rRNA genes might play less important roles in these species.

In fast-growing species, we indeed found a statistically significant negative correlation between genome size and rRNA gene positions (Fig I1; Spearman's ρ = -0.40, P = 0.005). In contrast, we did not find a significant correlation between genome size and tRNA gene positions (Fig I2; Spearman ρ = 0.04, P = 0.80). As expected, a positive correlation was found between genome size and the relative position ($position_{\mathrm{tRNA}} - position_{\mathrm{rRNA}}$) in fast-growing species (Fig I3; Spearman's ρ = 0.48, $P = 5.1\times10^{-4}$). We found no statistically significant correlations in slow-growing species ($P > 0.1$ for Fig I1-I3).

To control for the effects of genome size in our analysis of the dependence of genomic positions on μ_{max}, we analyzed linear regression models of the form

$$position \sim \log(\mu_{\mathrm{max}}) + genome\_size \, ,$$

where position is either $position_{\mathrm{tRNA}}$, $position_{\mathrm{rRNA}}$, or the relative position $position_{\mathrm{tRNA}} - position_{\mathrm{rRNA}}$. For fast-growing species, the inclusion of genome size as an additional regressor leads to small increases of R^2, from 0.32 to 0.36 (P = 0.07 for genome size) for rRNA position, and from 0.13 to 0.15 (P = 0.24 for genome size) for tRNA position. Thus, the maximal growth rate remains the main predictor of the two positions, with only minor contributions by genome size.

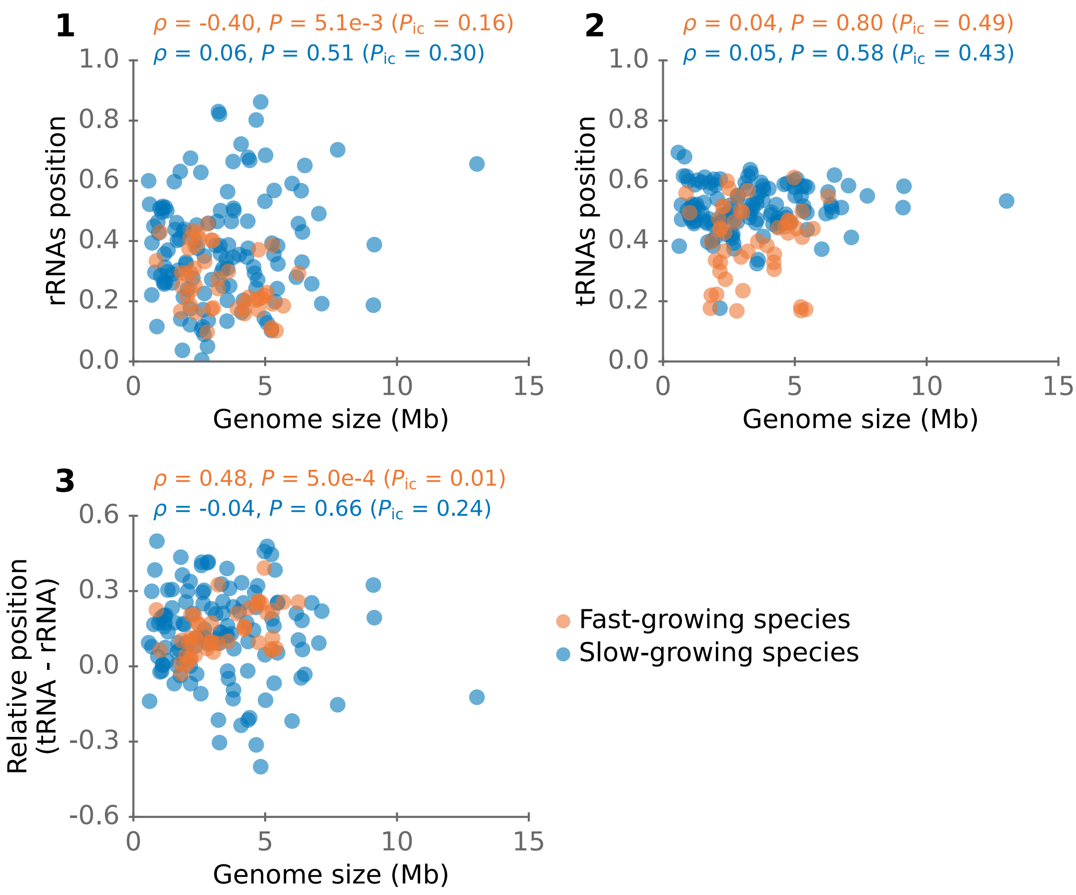

Fig I. The relationship between genome size and the positions of tRNA and rRNA genes. (panel 1) In fast-growing species, rRNA genes in larger genome tend to be closer to oriC. **(panel 2)** tRNA position is not correlated with genome size in both slow- and fast-growing species. **(panel 3)** In fast-growing species, the relative position ($position_{tRNA}$– $position_{rRNA}$) is positively correlated with genome size, indicating that gene dosage plays a more important role in establishing the optimal tRNA/ribosome expression ratio in these species.

Text C. The positions of tRNA genes are not correlated with the corresponding codon frequencies.

In the main text, we showed that the genomic averages of tRNA gene positions are affected by the maximal growth rate of a species (Fig 3B) and constrained by the optimal RNA growth law, which posits that tRNA genes are on average farther from oriC than rRNA genes (equation (1) and Fig 3C). When calculating the tRNA/rRNA gene dosage ratio (Fig 3D), we treated all tRNAs equally. However, tRNAs decode codons with different frequencies. While the optimal scaling of the tRNA/rRNA expression ratio (equation (2)) with growth rate is independent of codon frequencies, it is still conceivable that selection pressure toward specific genomic positions is stronger for tRNA genes whose products decode more abundant codons. We thus asked if there is a systematic dependence of genomic positions on the frequencies of the cognate codons.

As the anticodons of tRNAs are not annotated in RefSeq for some species [8], we identified the anticodon using tRNAscan-SE 2.0 [9] and used the wobble paring rule to find the cognate codon(s) of a given tRNA. Then, a tRNA's cognate codon frequency was calculated as the summed frequencies of all its cognate codon(s).

We tested if there is a correlation between a tRNA's position and its cognate codon frequency in a given species. Please note that the analysis here is different from Fig 3B in the main text. In the main text, we tested if the average position of tRNA genes tends to be close to oriC in fast-growing species. Here, we test if individual tRNAs with higher codon frequencies tend to be located closer to oriC in a given genome.

Statistically significant correlations ($P < 0.05$) between tRNA genomic position and the cognate codon frequency were found in 21 out of 170 species (Fig J1). 6 out of the 21 species show positive correlations, while the remaining 15 species show negative correlations. This means that in only 15 species those tRNAs that can decode more codons (by codon frequency) tend to be located closer to the origin of replication.

We also tested for a correlation between tRNA copy number (the number of tRNA genes with the same anticodon) and cognate codon frequency. We found that tRNAs with higher copy numbers tend to have higher cognate codon frequencies in most fast-growing species (Fig J2; $P < 0.05$ in 45 out of 48 species).

We thus conclude that in fast-growing species, the copy number of a tRNA gene but not its genomic position is strongly affected by its cognate codon frequency; this finding supports our equal treatment of tRNAs when calculating tRNA gene dosage.

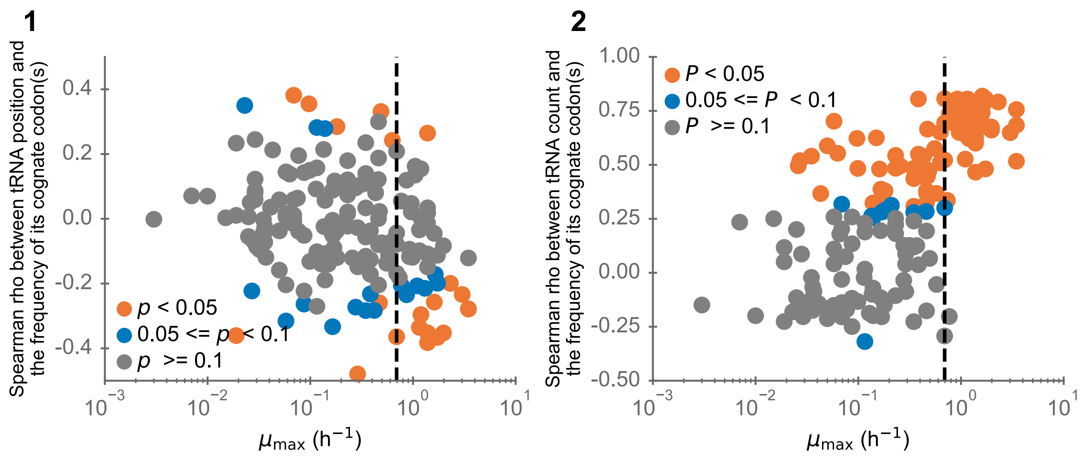

Fig J. Copy numbers of tRNA genes but not their positions are correlated with codon frequency. Each datapoint represents Spearman's rank correlation coefficient ρ for a single genome. Point color indicates statistical significance. (**panel 1**) Correlation between tRNA position and cognate codon frequency. (**panel 2**) Correlation between tRNA genomic copy number and cognate codon frequency.

Text D. The effect of assuming a constant TC/ribosome expression ratio instead of an optimal ratio

Several recent modeling studies have assumed that the TC/ribosome expression ratio is constant [4,12–16]. In contrast, we found that the optimal TC/ribosome expression ratio is growth rate-dependent (equation (1)), a relationship consistent with experimental data across species (Fig 1B and Fig 2A). In this section, we estimate by how much growth rate predictions are expected to change when accounting for this growth rate dependence. To be independent of any specific model, we estimated the associated change in the cellular cost for translation, where – consistent with the assumptions of our optimality estimate, equation (1) – we used the cytosol density as a proxy for cost; detailed predictions derived from this cost measure have been found to be in good agreement with experimental data for the *E. coli* translation machinery [17].

We first calculated the optimal dry mass per volume occupied by ribosomes and TCs as a function of growth rate according to our model. Assuming a constant protein concentration across conditions in *E. coli*, we first calculated the molar concentrations of ribosome and TC based on equations (9) and (10), respectively.

For comparison, we then calculated the concentrations of ribosome and TC under the constant assumption with $\mu \cdot [P] = k_{eff} \cdot [R]$, where μ is the growth rate, $\cdot[P]$ is the protein concentration, $[R]$ is the ribosome concentration, and k_{eff} is the effective turnover rate of the ribosome. The constant assumption assumes that the rRNA:tRNA mass ratio is 86:14 [12], resulting in a constant TC:ribosome molar ratio of 9.6:1. Further, $k_{eff} = k_{cat} \cdot K_M / ([TC] + K_M)$, with k_{cat} (22 s^{-1}) the ribosome turnover rate, $[TC]$ = 9.6 [R] the concentration of TC, and K_M = 122 µM the Michaelis constant (see Methods). The ribosome concentration $[R]$ can then be obtained by solving $\mu \cdot [P] = k_{eff} \cdot [R]$.

Using the respective molecular weights, we converted both estimates for the ribosome and TC concentrations into a combined cytosolic mass density of the core translation components. Comparing these combined mass densities, we found that the total dry mass allocated to translation is very similar between the two calculations at high growth rates; substantial differences are only found at low growth rates (Fig K).

Thus, the assumption of a constant TC/ribosome expression ratio will lead to very similar growth rate predictions at moderate to fast growth rates. However, translation is a very expensive process for fast-growing cells, and even these small differences will have a substantial effect on evolution in natural populations. The efficiency of natural selection in large bacterial populations likely explains why the optimal expression of the ribosome and TC (Fig 1B and Fig 2) and the differential genomic position of rRNA and tRNA genes (Fig 3) are consistent with experimental data across species.

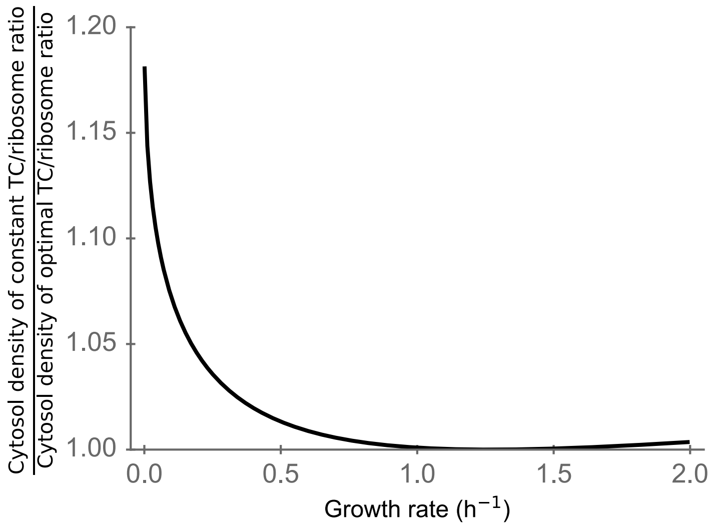

Fig K. Cost difference (in terms of dry mass density) between the optimal TC/ribosome expression ratio and a constant TC/ribosome expression ratio in *E. coli* as a function of growth rate.

References

1. Pine MJ. Steady-state measurement of the turnover of amino acid in the cellular proteins of growing Escherichia coli: existence of two kinetically distinct reactions. J Bacteriol. 1970;103: 207–215. doi:10.1128/jb.103.1.207-215.1970

2. Pine MJ. Regulation of Intracellular Proteolysis in Escherichia coli. J Bacteriol. 1973;115: 107–116.

3. Calabrese L, Grilli J, Osella M, Kempes CP, Lagomarsino MC, Ciandrini L. Role of protein degradation in growth laws. bioRxiv. 2021; 2021.03.25.436692. doi:10/gkmtmz

4. Klumpp S, Scott M, Pedersen S, Hwa T. Molecular crowding limits translation and cell growth. Proc Natl Acad Sci U S A. 2013;110: 16754–9. doi:10.1073/pnas.1310377110

5. Bobay L-M, Ochman H. Factors driving effective population size and pan-genome evolution in bacteria. BMC Evol Biol. 2018;18: 153. doi:10/gfjjbp

6. Atlung T, Hansen FG. Low-Temperature-Induced DnaA Protein Synthesis Does Not Change Initiation Mass in *Escherichia coli* K-12. J Bacteriol. 1999;181: 5557–5562. doi:10/gkznd8

7. Slager J, Kjos M, Attaiech L, Veening J-W. Antibiotic-Induced Replication Stress Triggers Bacterial Competence by Increasing Gene Dosage near the Origin. Cell. 2014;157: 395–406. doi:10.1016/j.cell.2014.01.068

8. Pruitt KD, Tatusova T, Brown GR, Maglott DR. NCBI Reference Sequences (RefSeq): current status, new features and genome annotation policy. Nucleic Acids Res. 2012;40: D130-5. doi:10.1093/nar/gkr1079

9. Chan PP, Lin BY, Mak AJ, Lowe TM. tRNAscan-SE 2.0: Improved Detection and Functional Classification of Transfer RNA Genes. bioRxiv. 2021; 614032. doi:10.1101/614032

10. Michelsen O, Teixeira de Mattos MJ, Jensen PR, Hansen FG. Precise determinations of C and D periods by flow cytometry in Escherichia coli K-12 and B/r. Microbiology. 2003;149: 1001–1010. doi:10.1099/mic.0.26058-0

11. Zheng H, Bai Y, Jiang M, Tokuyasu TA, Huang X, Zhong F, et al. General quantitative relations linking cell growth and the cell cycle in Escherichia coli. Nat Microbiol. 2020;5: 995–1001. doi:10/ggwqth

12. Bremer H, Dennis PP. Modulation of Chemical Composition and Other Parameters of the Cell at Different Exponential Growth Rates. EcoSal Plus. 2008;3: 765–77. doi:10.1128/ecosal.5.2.3

13. Scott M, Gunderson CW, Mateescu EM, Zhang Z, Hwa T. Interdependence of cell growth and gene expression: origins and consequences. Science. 2010;330: 1099–102. doi:10.1126/science.1192588

14. O'Brien EJ, Lerman J a, Chang RL, Hyduke DR, Palsson BØ. Genome-scale models of metabolism and gene expression extend and refine growth phenotype prediction. Mol Syst Biol. 2013;9: 693. doi:10.1038/msb.2013.52

15. Bosdriesz E, Molenaar D, Teusink B, Bruggeman FJ. How fast-growing bacteria robustly

tune their ribosome concentration to approximate growth-rate maximization. FEBS J. 2015;282: 2029–2044. doi:10.1111/febs.13258

16. Dai X, Zhu M, Warren M, Balakrishnan R, Patsalo V, Okano H, et al. Reduction of translating ribosomes enables *Escherichia coli* to maintain elongation rates during slow growth. Nat Microbiol. 2016;2: 16231. doi:10.1038/nmicrobiol.2016.231

17. Hu X-P, Dourado H, Schubert P, Lercher MJ. The protein translation machinery is expressed for maximal efficiency in Escherichia coli. Nat Commun. 2020;11: 5260. doi:10.1038/s41467-020-18948-x
