# Supplementary material for: An optimal growth law for RNA composition and its partial implementation through ribosomal and tRNA gene locations in bacterial genomes
Source: PLoS Genet. 2021 Nov 29;17(11):e1009939. doi: 10.1371/journal.pgen.1009939 (PMC8659690; doi:10.1371/journal.pgen.1009939)
Supplement: Attachment — Submitted filename: Reply2Reviewers.pdf [file pgen.1009939.s007.pdf]

Reviewer #1

The study suggests a strong relation between tRNA/rRNA ratio with growth rate. They suggest that this relation is under selection via the fact that rRNA genes are typically closer to the origin of replication than tRNA genes and thus have increasingly higher gene dosage at faster growth. The study is potentially interesting but as explained below there are various points that should be improved and it is hard for me to interpret the results without this additional info.

1) *abstract: "At the highest growth rates in E. coli, the tRNA/rRNA ratio appears to be regulated entirely through this effect." I do not see a support to this claim in the paper. The highest correlation reported in the paper is lower than 0.6 (related to 36% explained variance).*

Response: We apologize for not making this statement sufficiently clear. The tRNA/rRNA dosage ratio is almost identical to the tRNA/rRNA expression ratio at the highest growth rates in *E. coli* (Fig 1B), indicating that no other regulatory mechanisms are required to achieve this expression ratio at maximal growth.

Action: We have revised the sentence to clarify our claim (line 20).

2) *It will be interesting to show that the observed signal is correlated with selection pressure (e.g. effective population size).*

Response: A recent paper by Bobay and Ochman (*BMC Evol Biol* 18, 153 (2018)) lists effective population sizes (N_e) for 46 out of the 170 species in our dataset. As expected, N_e is correlated with genome size and growth rate (Panel 4 and 5 in Fig D in S1 File, copied below). However, in separate analyses for fast-growing and slow-growing species, we found no significant correlations between N_e and the position measures analyzed in our manuscript (position of rRNA genes, position of tRNA genes, and relative position $position_{tRNA}$ – $position_{rRNA}$; Panels 1-3 in Fig D in S1 File, copied below). In all cases, a Spearman rank correlation test resulted in P>0.1. Thus, there is no clear connection of the observed signals to the strength of selection acting on the respective species (as indicated by N_e).

Action: We discuss the lack of a correlation with the effective population size (line 233) and provide a corresponding supporting figure (Fig D in S1 File), copied below.

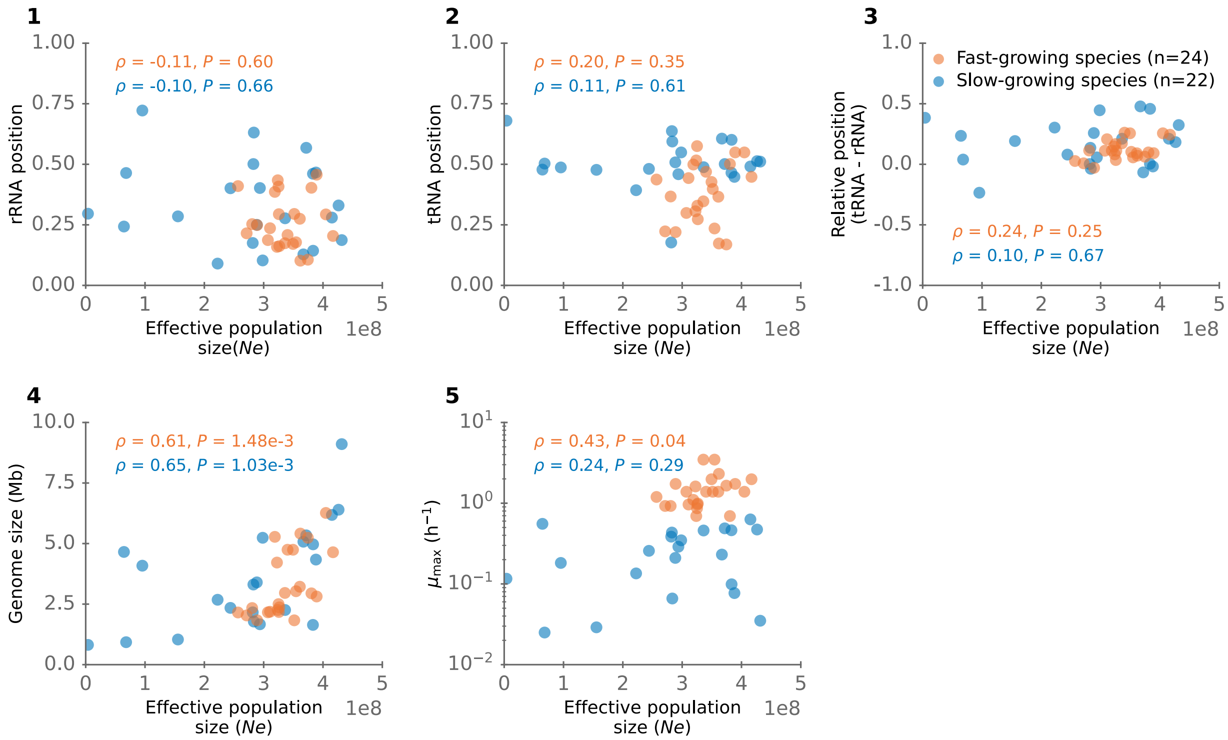

Fig D. Effective population size (*N*e) is not correlated with the positions of tRNA and rRNA genes. The effective population sizes for 46 out of the 170 species were obtained from Ref. [5]. As shown in **panels 1-3**, we found no statistically significant Spearman rank correlations ($P > 0.1$) between N_e and the different positions considered in our study (position of rRNA genes, tRNA genes, and the relative position, $position_{tRNA} - position_{rRNA}$) when analyzing fast- and slow-growing species ($P > 0.1$, see colored text in panels 1-3). In contrast, we found statistically significant correlations between N_e and genome size (**panel 4**) and, for fast-growing species, between N_e and maximal growth rate μ_{max} (**panel 5**).

3) *The paper focus on the tRNA/rRNA ratio. Does this ratio correlate with the number of tRNA genes and/or the number of rRNA genes in the genome?*

Response: Yes, in both fast-growing and slow-growing species, both the tRNA/rRNA gene dosage ratio and the tRNA/rRNA genomic ratio (gene copies per genome) are negatively correlated with the number of tRNA and with the number of rRNA genes in the genome (Fig E in S1 file, copied below, panels 3-6; *P*-values based on phylogenetically independent contrasts are shown in parentheses).

Moreover, fast-growing species have more tRNA and rRNA genes (panels 1 and 2). While a negative correlation between the tRNA/rRNA ratios and the number of rRNA genes would also occur if the numbers of tRNA and rRNA genes were independent, a negative correlation of the tRNA/rRNA ratios with the number of tRNA genes would not be expected in this case; the latter thus provides strong support for our hypothesis.

Action: In the revised manuscript, we briefly discuss the relationships between the absolute numbers of tRNA and rRNA genes with the maximal growth rate and with the ratio between these

numbers (tRNA/rRNA genomic and gene dosage ratio) in slow vs. fast-growing species (Line 250, line 259, and Fig E in S1 file).

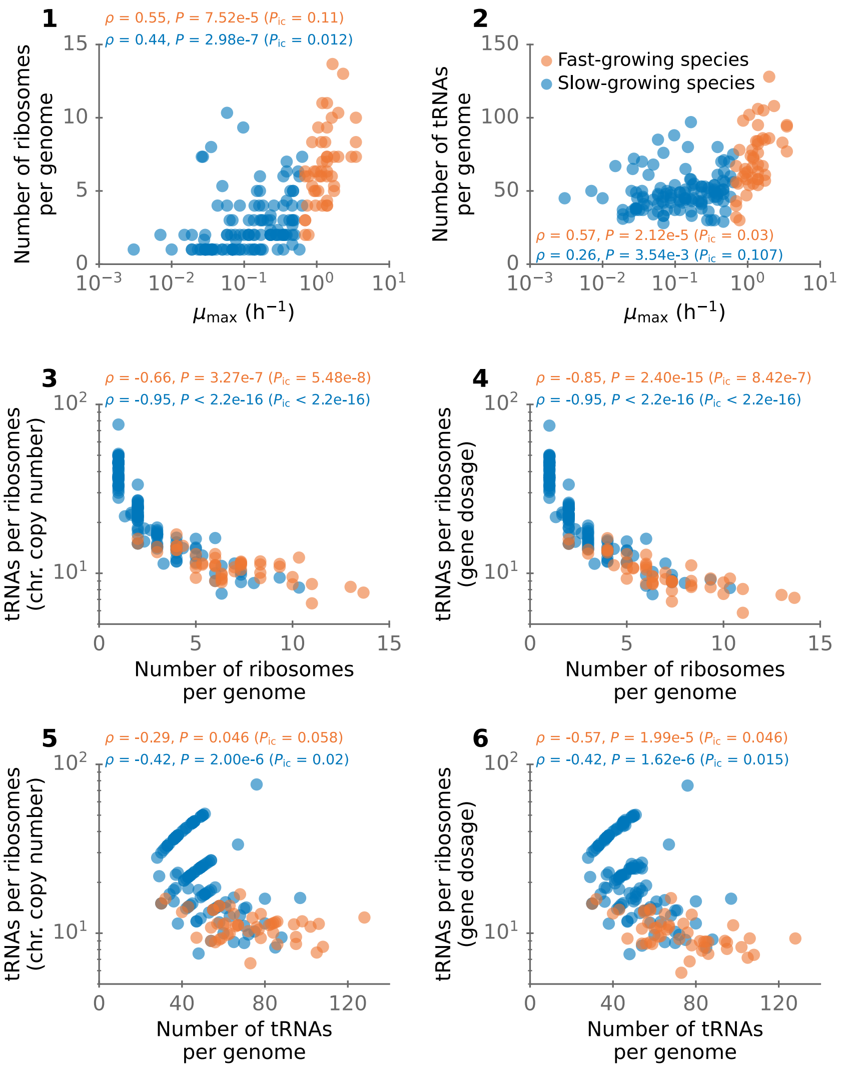

Fig E. The numbers of genomically encoded rRNA genes and tRNA genes are positively correlated with µmax and negatively correlated with tRNA/ribosome ratios. With increasing maximal growth rate μ_{max}, bacterial genomes harobr more rRNA genes (**panel 1**) and tRNA genes **(panel 2). Panels 3-6** show the relationship between tRNA/ribosome dosage and genomic ratios and the number of ribosome and tRNA genes in the genome. While a negative correlation between the tRNA/ribosome ratios and the number of rRNA genes would trivially occur also if the numbers of tRNA and rRNA genes were independent (**panels 3 and 4**), a negative correlation of the tRNA/ribosome ratios with the number of tRNA genes would not be expected in this case (**panels 5 and 6**); the latter observation thus provides strong support for our hypothesis that the relative number of tRNA and rRNA genes are constrained to optimize translation efficiency.

4) *How is the reported relation related to the length of the genome.*

Response: As suggested by the reviewer, genome size influences the position of rRNA genes, at least in fast-growing species (Fig G in S1 text, copied below). At the same DNA replication rate, smaller genomes need less time to replicate than larger genomes. Thus, at the same growth rate, bacteria with smaller genomes are expected to have fewer replication forks in the cell. For example, no multiple replication rounds were observed in fast-growing *Streptococcus pneumoniae*, which has a 2.1 Mb genome (Slager 2014, Cell 157:395). This indicates that replication-associated gene dosage effects may be less important in species with smaller genomes. Consistent with this notion, we found a statistically significant negative correlation between genome size and rRNA gene position in fast-growing species (panel 1); in fast-growing species, we also found a positive correlation between genome size and the relative position (tRNA position – rRNA position) (panel 3). However, we did not find a correlation between tRNA position and genome size (panel 2). No statistically significant correlations were found in slow-growing species.

Action: We now discuss the effects of genome size on tRNA and rRNA positions in supporting information (Text A in S1 File, which includes the figure below) and briefly discuss it in the main text (line 223).

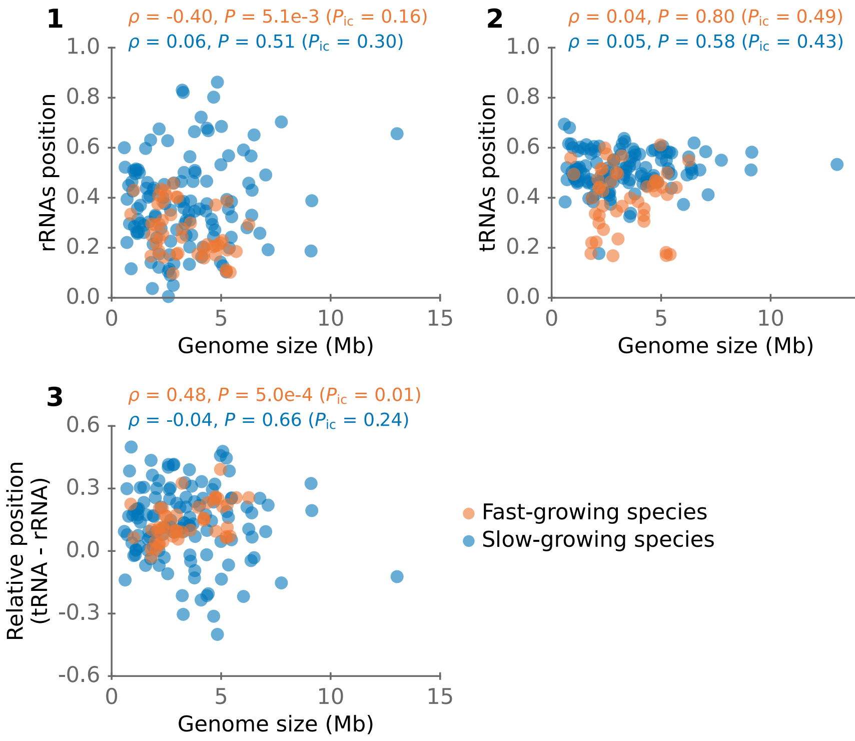

Fig G. The relationship between genome size and the positions of tRNA and rRNA genes. (panel 1) In fast-growing species, rRNA genes in larger genome tend to be closer to oriC. **(panel 2)** tRNA position is not correlated with genome size in both slow- and fast-growing species. **(panel 3)** In fast-growing species, the relative position ($position_{tRNA}$– $position_{rRNA}$) is positively correlated with genome size, indicating that gene dosage plays a more important role in establishing the optimal tRNA/ribosome expression ratio in these species.

5) *"we here analyze a coarse-grained translation model that only considers peptide elongation, where the active ribosome acts as an enzyme that converts ternary complexes (TC), consisting of elongation factor Tu (EF-Tu), GTP, and charged tRNA, into an elongating peptide chain following Michaelis-Menten kinetics .." This model includes many extreme approximations of the translation process (e.g. it does not include traffic jams of ribosomes, their movements, number of ribosomes and tRNAs in the cell, etc). You should evaluate the bias due to these approximations and show that they do not bias the conclusions*

Response: Translation is indeed a very complicated process. From a modeling point of view, it could be described with details of all sub-processes of initiation and elongation, with parameters based on experimental estimates in specific conditions (Rudorf *et al.* 2014, Plos Comput Biol 10: e1003909); or it could be modeled as a totally asymmetric simple exclusion process (TASEP), with parameters derived from ribosomal profiling data (Erdmann-Pham *et al.* 2020, Cell Syst 10: 183). Following earlier work (Klumpp *et al.* 2013, PNAS 110: 16754), we chose an alternative, coarse-grained approach, where ribosomes are treated as enzymes and ternary complexes as substrates. This coarse-grained translation model is able to estimate how many ribosomes and ternary complexes (tRNA and EF-Tu) are required in the cell, based on the ribosomes' average turnover number for elongation and their average affinity for ternary complexes. While the model does not account explicitly for ribosomal traffic jams, their existence can be integrated into a coarse-grained model through its influence on the turnover number.

We recently used a more detailed model similar in spirit to the one examined here, which shares many of the simplifications of the current model (e.g., ignoring traffic jams and movements of ribosomes), to provide accurate quantitative predictions of cellular resource allocation into the different components of the translation machinery (Hu et al. 2020, Nature Communications 11: 5260). For the purpose of the current work – an approximation to the total required concentrations or rRNA and tRNA at different growth rates – we consider the simple, coarse-grained model to be the most adequate representation of translation.

Action: We now discuss the simplifications of the coarse-grained model compared to the actual process of translation explicitly in the text (line 279).

6) *"For six out of the seven datasets in Fig. 1, the tRNA/ribosome ratio decreases with increasing growth rate. The only exception, the cyanobacterium Synechococcus elongatus, has a much smaller maximal growth rate (μ_{max}=0.23h^{-1}) than the other species, and its tRNA/rRNA ratio does not show a clear growth rate dependence [48]. "Moreover, a strong selection pressure towards optimal tRNA/ribosome ratios appears to exist in fast-growing bacteria (Fig 1b)." All claims should be followed by p-values. This is a general comment related to all results in the paper.*

Action: As suggested by the reviewer, we added *p*-values where appropriate (e.g., line 173, 210, 211, 245, and 249).

7) *Figure 1: the theoretical graphs are not so close to the empirical points. You should at least report a quantitative measure relating to the fit of the theoretical graphs the empirical points and prove that it is significant.*

Response: As seen from Figure 1B, there is a large variation across different experimental datasets for *E. coli*, complicating the interpretation of the quantitative agreement between theory and experiment. We quantified this agreement through two measures: the normalized root-mean-square error, NRMSE (= RMSE divided by the observed mean), and the Pearson correlation between data and predictions (Fig. 1B and Fig. 2A). The correlation between the experimental data and the fitted line for *E. coli* (Fig. 1B) is $r^2 = 0.50$; please note that we did not fit any parameters to obtain this agreement. Squared correlation coefficients for the other species (Fig. 2A) range from 0.67 to 0.97; these all include one fitting parameter (k_{cat}).

Action: We added the requested quantifications to Fig 1B and Fig 2A.

8) *The reported p-values (related to the correlations) assume that the points are independent. However, this is not the case for organisms (that were evolve from a common ancestor). You should correct for this when you compute p-values (there are approaches that consider the evolutionary tree when computing the correlations and p-values).*

Response: We thank the reviewer for reminding us of the non-independence of the species data. All the previously reported statistically significant correlations are still statistically significant when performing the analysis on Felsenstein's phylogenetically independent contrasts; the only exception is the negative correlation between growth rate and tRNA position, which now became statistically significant also in slow-growing species (N=122).

Action: We now perform all statistical tests across genomes using Felsenstein's phylogenetically independent contrasts and reported the corresponding p-values following the original p-values in the text and figures. The methods used for these statistical calculations are reported in the Methods (line 376).

9) *It will be helpful to see all the information related to the distributions of distances of rRNA genes and tRNA from origin of replications in the analysed organisms.*

Action: The positions of individual tRNA and rRNA genes in *E. coli* are now shown in Fig C in S1 File (copied below), with the mean genomic position 0.20 ± 0.17 (mean ± standard deviation) for rRNA genes and 0.45 ± 0.27 for tRNA genes (also reported in line 144). The positions for the other species are provided in S3 Table.

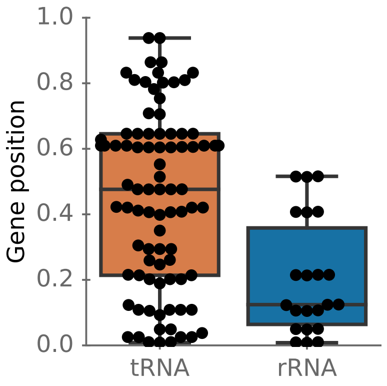

Fig C. Gene positions of rRNA and tRNA genes in E. coli. rRNA genes are located closer to oriC than tRNA genes in *E. coli*, with genomic position 0.20 ± 0.17 (mean ± standard deviation) for rRNA genes and 0.45 ± 0.27 for tRNA genes.

10) "Moreover, we found that both rRNA and tRNA genes tend to be located ever closer to oriC with increasing μ_{max} (Spearman's rank correlation coefficient between μ_{max} and position rRNA: = −0.59, = 9.2 × 10/1; between µmax and position tRNA: = −0.40, = 0.0047). .."
Please provide all the dot plots related to the correlations. Do you control for the differences in the size of the genomes among the analysed organisms and the number of oriC?

Response: (a) dot plots: We agree that showing the underlying dot plots is a good idea.

(b) genome sizes: As already stated in our response to the reviewer's comment 4, genome size is correlated with rRNA position in fast-growing species. To control for the effects of genome size, we thus analyzed linear regression models of the form

position ~ log(μ_{max}) + genome_size

For fast-growing species, the inclusion of genome size as an additional regressor lead to an increase of R^2 from 0.32 to 0.36 (p=0.07 for genome size) for rRNA position, and from 0.13 to 0.15 (p=0.24 for genome size) for tRNA position. Thus, the maximal growth rate remains the main predictor of the two positions, independent of genome size.

(c) number of oriC: In our dataset, 35 out of 170 species have 2 oriCs (the remainder has 1 oriC). In each case, the two oriCs are very close to each other on the chromosome, with a maximal normalized chromosomal distance of 0.0035, i.e, 0.35% of half the circular genome length (the maximal physical distance between two oriCs is 2.7 kb). Thus, the existence of multiple oriCs in some species does not bias our results.

Action: (a) The dot plots are now shown in Fig 3A and 3B; Fig. 3C-3E show dot plots for the other correlations reported.

(b) In the revised manuscript, we discuss the potential influence of genome size and provide the results from the regression models (line 223 and Text A in S1 File).

(c) We now mention the existence of two oriC in some species and their close positioning in the main text (line 371 in Methods).

11) Your model assumes that all the tRNA are identical while eventually they decode codons with different frequencies in the transcriptome and AA with different frequencies in the proteome. Do you see differences in terms of the distances of the different tRNA from oriC?

Response: There is no correlation between the position of a tRNA and its codon frequency in most species. For a specific tRNA in a given genome, we identified the anticodons by tRNAscan-SE 2.0 and used the wobble paring rule to find the cognate codon(s) (codons that can be translated by this tRNA). Then, the frequency of a tRNA's cognate codon(s) was calculated as the summed frequency of all its cognate codon(s). We then performed correlation tests between tRNA positions and the associated cognate codon frequencies in a given species.

As expected, the <u>number</u> of tRNA genes per tRNA species showed a positive correlation with the genomic frequencies of the corresponding codons for practically all fast-growing species ($p < 0.05$ in 45 out of 48 species), and also for 37 out of 122 slow-growing species (panel 2 of Fig H in S1 file, copied below). For most fast-growing species, we also found a negative correlation between the <u>positions</u> of tRNA genes and the corresponding codon frequencies (35 out of 48); for 12 of these, the correlation was statistically significant ($p<0.05$). Overall, we found statistically significant negative correlations ($p < 0.05$) between tRNA genomic positions and the frequencies of cognate codon(s) in only 15 out of the 170 species analyzed (panel 1).

However, it is not obvious to us which pattern to expect: the optimal dosage of all tRNAs should increase in sync with growth rate, so that the optimal genomic position would in fact be identical for codons with high and low frequencies. However, natural selection may act more strongly on those tRNA genes that encode more abundant tRNAs, potentially explaining the observation of a negative correlation in most fast-growing species.

Action: We briefly report these analyses in line 298 and more extensively in Text B in S1 File.

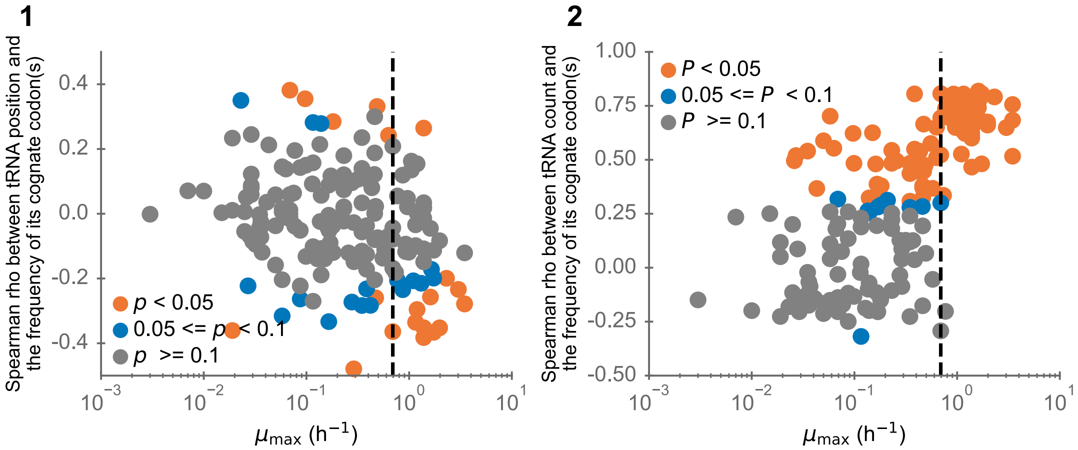

Fig H. Copy numbers of tRNA genes but not their positions are correlated with codon frequency. Each datapoint represents Spearman's rank correlation coefficient ρ for a single genome. Point color indicates statistical significance. (**panel 1**) Correlation between tRNA position and cognate codon frequency. (**panel 2**) Correlation between tRNA genomic copy number and cognate codon frequency.

12) how do you decide on the treshold of slow and high growth rate?

Response: The threshold (doubling time 1 h) is roughly the sum of the replication time (C≈40 min) and division time (D≈20 min) in *E. coli*. Species with minimal doubling times lower than this threshold are very likely to have multiple replication rounds per cell cycle; accordingly, replication-associated gene dosage effects are expected to be strongest in these fast-growing species.

Action: We now explain the rationale for our choice of threshold in the text (line 194).

13) "While slowly growing species show a wide range of tRNA/ribosome gene dosage ratios, the ratio in fast-growing species shows a much tighter distribution." This may be related to tighter selection for fast growing bacteria related to other (e.g. genome size, tRNA copy numbers, etc)

Response: (**a**) Genome size. As we discussed in response to the reviewer's comment 4 (and in Text A in S1 File), genome size does affect the position of genes in fast-growing species; the likely reason is that the replication-associated gene dosage effect is more relevant in large genomes. However, the genome size itself seems not to be under tight selection in fast-growing species, as fast-growing species do not show a very narrow range of genome sizes (see the dotplot below).

(**b**) tRNA and rRNA gene copy number. The tRNA/ribosome gene dosage ratio is of course not independent from the number of rRNA and tRNA genes (see Fig E in S1 file, copied above in response to the reviewer's comment 3). Our point is that not only the absolute number (copy per genome) of rRNA and tRNA genes, but also the relative number of rRNA and tRNA genes (tRNA/rRNA genomic ratio and gene dosage ratio) is under tighter selection in fast growing species. As translation is a very expensive process in fast-growing species, tRNA and ribosome are expressed near the optimal ratio across growth rates (Fig. 1B and Fig. 2A). This selection can be also seen on the tRNA/ribosome genomic ratio (Fig. 3E) and gene dosage ratio (Fig. 3D) in fast-growing species.

Action: The contribution of genome size to gene positions is discussed in Text A in S1 File and summarized following line 227. The tighter selection on the absolute number of genes and relative number of genes is discussed in line 250 and 259.

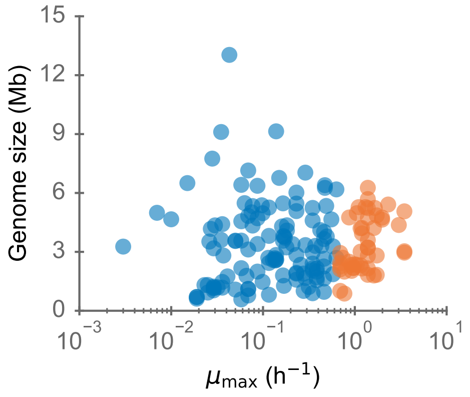

Relationship between genome size and maximal growth rate.

Reviewer #2

In this work, the Authors propose an optimality principle for the expression of ribosomes and associated proteins across organisms, and investigate the role of gene concentration in setting the correct tRNA/rRNA ratio. I was surprised by the short length of the manuscript, but the Authors were able to make their points clearly and concisely. The manuscript could be improved upon by providing some additional comments and analysis, which I list below.

1. *About the optimality principle: how do the predictions compare to other proposed explanation of the tRNA/ribosome ratio, e.g. based on molecular crowding (Klumpp et al., PNAS 2013)? The proposed principle would gain a lot of credibility if alternative hypothesis did a worse job at explaining the data.*

Response: Klumpp *et. al* found that the binding of the ribosome and the ternary complex (TC, complex of charged-tRNA, elongation factor Tu, and GTP) is limited by molecular crowding (which slows down the diffusion of the TC). They then estimated the optimal EF-Tu/ribosome ratio by maximizing the protein production rate given the proteome budget allocated to translation (ribosomal protein plus elongation factor Tu). The predicted EF-Tu/ribosome ratio was substantially lower than the measured data (Fig B in S1 file, copied below, data extracted from Fig. 4C of Stefan Klumpp's paper).

We predicted the tRNA/ribosome ratio in a similar way. However, we assume that it is the total dry mass contribution rather than the proteome fraction that best captures cellular investment; instead of maximizing the growth rate at a given protein budget, we minimize the combined dry mass concentration of ribosome and TC at the give protein production rate (the product of measured protein concentration and growth rate). The assumption of a parsimonious usage of dry mass concentration is based on the observation that *E. coli* has a limited and near constant cell density across conditions. Thus, if a certain proportion of cytosolic space is occupied by any one particular molecule, it will not be available for other molecules. In this sense, we incorporate the molecular crowding in the title of the Klumpp et al. paper more deeply into our model, by assuming that crowding (by proteins and functional RNA) not only influences the binding of the TC, but also constrains overall cellular activity, including the translation process.

The effectiveness of our assumptions has been discussed in our previous work (Hu et al. 2020, Nature Communications 11: 5260). As shown in the figure, the consideration of the total concentration of macromolecules (including both RNA and protein) predicts the tRNA/ribosome ratio much better than only considering protein cost (as done by Klumpp). In this context, it is important to note the mass fractions of protein in the ribosome and the ternary complex: protein accounts for roughly 1/3 of the ribosome mass, whereas it accounts for roughly 2/3 of the ternary complex mass. If only the protein cost is considered, the ternary complex appears much more expensive to the cell, resulting in a lower predicted ternary-complex/ribosome ratio.

Action: We now discuss these issues in the text (line 122) and in supporting information (Fig B in S1 File, copied below).

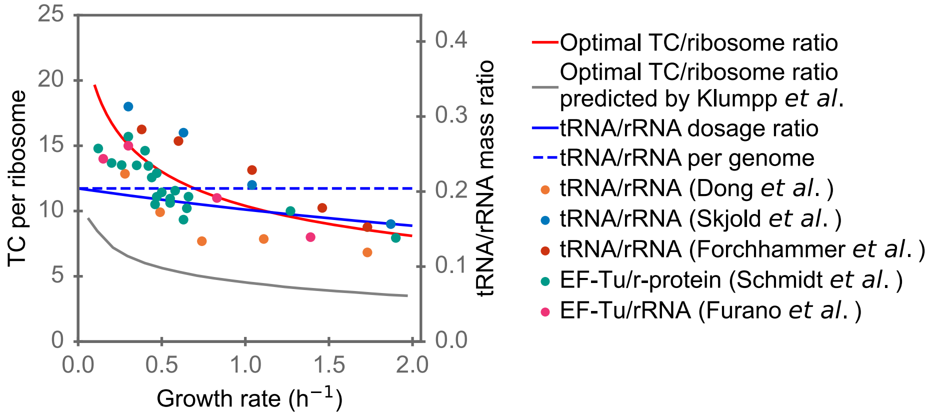

Fig B. Comparison of the optimal TC/ribosome expression ratio with a previously reported optimal TC/ribosome ratio for *E. coli* [4]. Klumpp *et. al* [4] predicted the optimal TC/ribosome expression ratio by identifying the proteome fractions of ribosome and TC that maximize growth rate in a coarse-grained, phenomenological model of cellular growth [4]. This optimal proteome allocation pattern results in a predicted TC/ribosome ratio (gray line, extracted from Fig 4C in [4]) that is substantially lower than the experimentally observed data. Here, we considered the optimal resource allocation into cellular dry mass (RNA and protein combined; red curve), motivated by the near-constant cellular dry mass density across growth conditions. The optimal dry mass allocation explains the experimentally observed TC/ribosome expression ratios much better than the optimal proteome allocation considered in Ref. [4]. Protein accounts for roughly 1/3 of the ribosome mass, whereas it accounts for roughly 2/3 of the ternary complex mass. If only the protein cost is considered, the ternary complex appears much more expensive to the cell, resulting in a lower predicted ternary-complex/ribosome ratio.

2. *Already in the abstract, the Authors state that "at the highest growth rates in E. coli, the tRNA/rRNA ratio appears to be regulated entirely" from the effect of varying gene dose across growth rates. However, the predicted effect is quite small (Fig. 1a): changing growth rate from 1/h to 2/h, the blue line changes by about 10%. The available experimental data seems too scattered to be able to confirm such tiny effect; the Authors should instead state that the data and the prediction are compatible, i.e. there is no necessity of additional regulation (although it cannot be excluded).*

Response: We apologize for not making this statement sufficiently clear. The tRNA/rRNA dosage ratio is almost identical to the tRNA/rRNA expression ratio at the highest growth rates in *E. coli* (new Fig 1B), indicating that no other regulatory mechanisms are required to achieve this expression ratio in this condition.

Comment: We have revised the sentence to clarify our claim (line 20).

3. *The Authors did not comment at all about the values of k fitted in Fig. 1b. Did you obtain any biological insight from the observed values of k? To validate the model proposed by the Authors, it would be interesting to compare the values of k=sqrt(k_cat K_M) fitted for the various organisms in Fig. 1b to values obtained from experimentally measured k_cat*

and K_M at least for some species. Such comparison would considerably strengthen the optimality principle argumented by the Authors.

Response: Motivated by the reviewer's comment, we examined equations (1) and (2) further, finding that mathematically identical versions can be formulated with only one species-specific parameter, the ribosomal turnover number k_{cat}. We then estimated these k_{cat} values by fitting the re-formulated equation (1) to the data points in Fig 1B and Fig 2A. In support of our model, we found that the fitted k_{cat} values are statistically significantly correlated with measured ribosome effective turnover number (k_{eff}) values.

More specifically: as the binding between the ribosome and the TC is limited by the diffusion of the TC, K_{m} can be approximated by $K_{\mathrm{m}} \approx k_{\mathrm{cat}}/k_{\mathrm{on}}^{\mathrm{diff}}$, with $k_{\mathrm{on}}^{\mathrm{diff}}$ the diffusion-limited binding rate (Klumpp *et al.* 2003, PNAS 110: 16754). Thus, $k \coloneqq \sqrt{k_{\mathrm{cat}} \cdot K_{\mathrm{m}}} \approx k_{\mathrm{cat}}\sqrt{1/k_{\mathrm{on}}^{\mathrm{diff}}}$. As the cytosolic density of macromolecules (and hence diffusion) is similar across microbes, we can assume that $k_{\mathrm{on}}^{\mathrm{diff}}$ is (approximately) a constant. Equation (1) and (2) only contain the species-specific parameter k_{cat} , and equation 1 can be rewritten as

$$\frac{[TC]}{[R]} = \frac{a \cdot k_{\mathrm{cat}}\sqrt{1/k_{\mathrm{on}}^{\mathrm{diff}}}}{\sqrt{a \cdot \mu \cdot [P]} + k_{\mathrm{cat}}\sqrt{1/k_{\mathrm{on}}^{\mathrm{diff}}}} \qquad (S1)$$

Reformulating equation (S1), we can get equation (S2), which is equivalent to equation (1) in the main text:

$$\frac{[TC]}{[R]} = \frac{a \cdot k_{\mathrm{cat}}}{\sqrt{a \cdot \mu \cdot [P] \cdot k_{\mathrm{on}}^{\mathrm{diff}}} + k_{\mathrm{cat}}} \qquad (S2)$$

We used least squares (Levenberg–Marquardt algorithm) to fit the measured data of each species (in Fig 1B and Fig 2A) to equation S1 to estimate k_{cat} for each species. To compare these estimates to independent experimental values, we estimated the effective ribosome turnover number (k_{eff}) by $\mu \cdot [P] = k_{\mathrm{eff}} \cdot [R]$, where μ is the growth rate, $[P]$ is the protein concentration, and $[R]$ is the ribosome concentration (data in S4 Table).

We exclude the species *Streptococcus pyogenes* in this analysis. Its tRNA/ribosome ratio decreases with increasing growth rate, which is consistent with our theory. However, our model depends on $k_{\mathrm{on}}^{\mathrm{diff}}$, which is assumed to be a constant on normal osmotic conditions. The tRNA/ribosome for *Streptococcus pyogenes* were measured under different osmotic pressures (Panos *et al.* 1965, Biochem Biophys Res Commun 19: 62). As the osmotic pressure affects the diffusion rate of all cytosol components, $k_{\mathrm{on}}^{\mathrm{diff}}$will also change for this species under the studied conditions, violating our assumptions. We also excluded *Synechococcus elongatus* from the fitting, as the data shows no significant correlation between growth rate and the tRNA/rRNA ratio.

The Pearson correlation between the k_{cat} values fitted according to our theory and k_{eff} estimated from independent experimental data is $r = 0.79$ ($P = 0.06$; see Fig 2B, copied below). Since both k_{cat} and k_{eff} estimates rest on (different!) simplifying assumptions, and because the tRNA/ribosome ratios used for fitting equation (S1) were measured with different methods by different groups, we

do not expect a perfect correlation; that we still get an r^2 of 62% appears to strongly support our analyses.

Action: We discuss these analyses in the main text (line 178, Fig 2B).

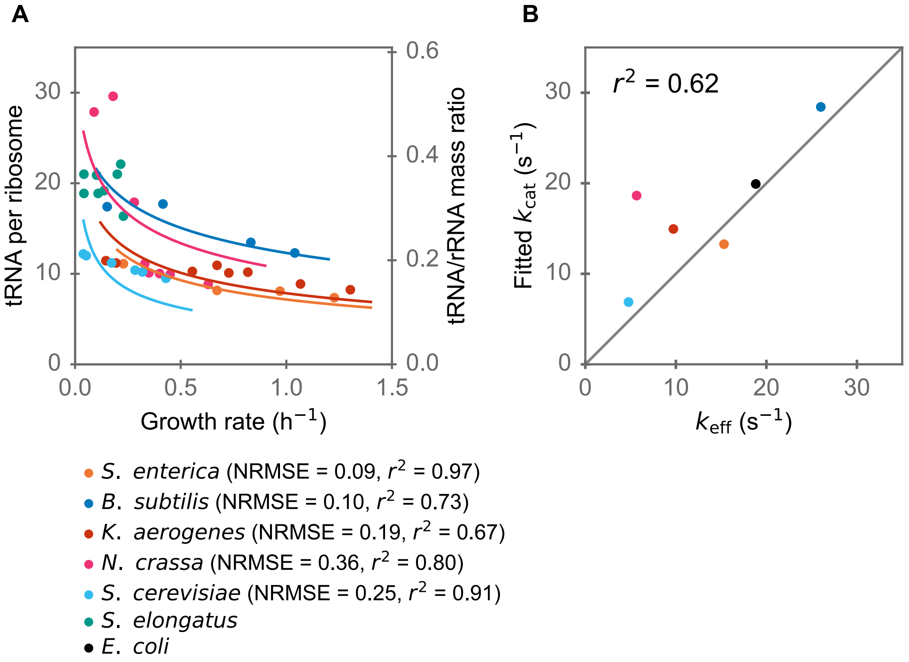

Fig 2. The RNA growth law across species. (A) Experimentally observed tRNA/ribosome expression ratios in different microbes decrease with growth rate, consistent with the predicted optimal tRNA/ribosome expression ratio. For each species except *S. elongatus*, which is a slow-growing species and shows no systematic growth rate dependence, we fitted equation (1) to the data by varying the single adjustable parameter k_{cat} (colored lines). Note that the y-axis on the right-hand side is based on the tRNA/rRNA mass ratio for bacteria. For eukaryotic microbes, the tRNA/rRNA mass ratio should be scaled by a factor of 0.84 according to equation (15). (B) Comparison of fitted k_{cat} and effective ribosome turnover number k_{eff}.

4. *Here below I have more technical comments:*

a) *Fig. 2e: The value of the C period is hardly known for most cells. The Authors make use of a reasonable estimate of C obtained dividing the size of the chromosome by a fixed replication rate k_rep. However, even Ref. [27] indicates that slow-growing organisms can have much slower replication rates. What would change in Fig. 2e if the k_rep increased with the maximum growth rate of the organisms varied across organisms?*

Response: Briefly: a species-specific replication rate does not appear to affect our conclusions. We searched the literature for replication rates of bacteria. In total, we found the replication rate (or C period) of 5 species in our dataset: 1 slow-growing species and 4 fast-growing species (S5 Table). We fitted μ_{max} (log scale) and replication rate (log scale) to a linear model (panel 1 of Fig I in S1 File, copied below) and used this linear model to estimate the μ_{max}-dependent DNA replication rate for all species in our dataset. The tRNA/ribosome dosage ratio estimated using this μ_{max}-dependent replication rate is very similar to that estimated with a constant replication rate (k = 1000 s^{-1}) (panel

2; R^2 = 0.973). Accordingly, the dependence of the tRNA/ribosome dosage on the maximal growth rate is very similar between the μ_{max}-dependent replication rate (Fig I3, $\rho = -0.47$, $P = 7.0 \times 10^{-4}$ for fast-growing species; $\rho = -0.47$, $P = 5.5 \times 10^{-8}$ for slow-growing species) compared to our original model using a constant replication rate (k = 1000 s^{-1}; Fig 3D) (slowly growing species: $\rho = -0.44$, $P = 2.8 \times 10^{-7}$; fast-growing species: $\rho = -0.49$, $P = 4.3 \times 10^{-4}$).

Action: We discuss these analyses in supporting information (Text C in S1 File, which we refer to in line 242 in the main text).

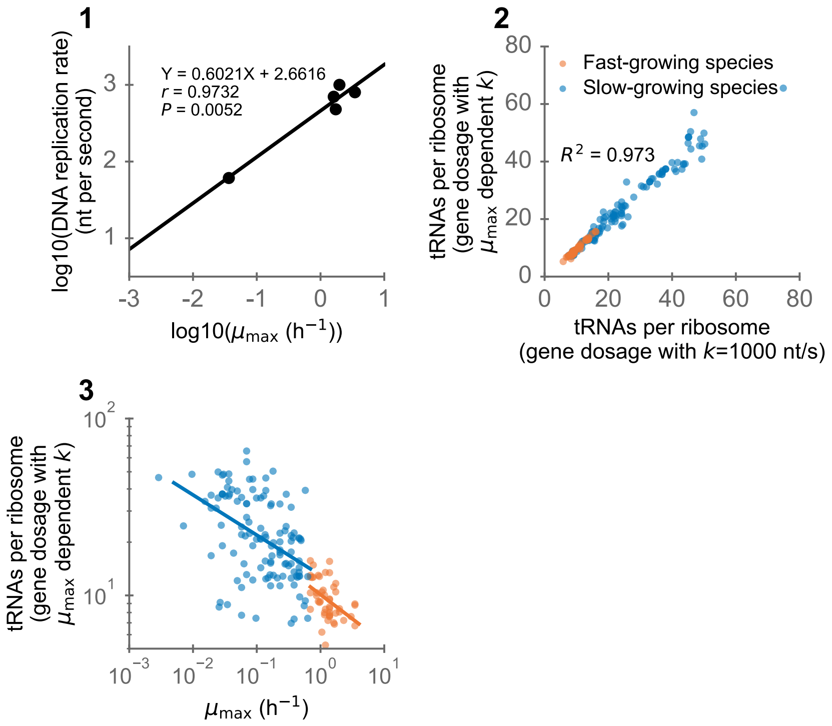

Fig I. The effect of a μ_{max}-dependent DNA replication rate on the tRNA/ribosome gene dosage ratio. (**panel 1**) linear regression on log-log scale to predict the DNA replication rate from μ_{max}. (**panel 2**) Comparison of tRNA/rRNA gene dosage ratios calculated using a constant DNA replication rate (x-axis) and using a μ_{max}-dependent replication rate. (**panel 3**) Relationship between the tRNA/rRNA gene dosage ratio calculated using a μ_{max}-dependent replication rate and μ_{max}.

b) *In the derivation of Eq. (2), the authors equate the protein synthesis flux to the product of the growth rate and the protein concentration. While this should be mostly fine for fast growing cells, I wonder what impact protein degradation has on the results. How would the fitted lines change by assuming a nonzero protein degradation rate?*

Response: Protein degradation will have a noticeable effect on the optimal tRNA/ribosome ratio only at growth rates that are so low that the protein degradation rate becomes comparable to the growth rate. The protein degradation function can be written as $1 - f_{deg} = e^{-k_{deg} \cdot t}$, in which f_{deg} is the fraction of degraded protein within time t and k_{deg} is protein degradation rate. With this equation, k_{deg} was estimated to range from 0.005 h^{-1} to 0.04 h^{-1} based on data reported in (Pine 1973, J Bacteriol 115: 107), and to range from 0.025 h^{-1} to 0.03 h^{-1} with data reported in (Pine 1970, J Bacteriol 103: 207). To be conservative, we chose k_{deg} = 0.04 h^{-1}, the largest reported degradation

rate, in our analysis. As degradation rate is relatively small compared to the maximal growth rate of *E. coli*, protein degradation has a very small effect on the optimal TC/ribosome ratio even at the lowest growth rates observed for *E. coli* (see Figure A in S1 File, copied below).

Action: We discuss these issues in supporting information (Fig A in S1 File) and mention the likely relevance of protein degradation for species with maximal growth rates on the same order of magnitude as protein degradation rates in the main text (line 117).

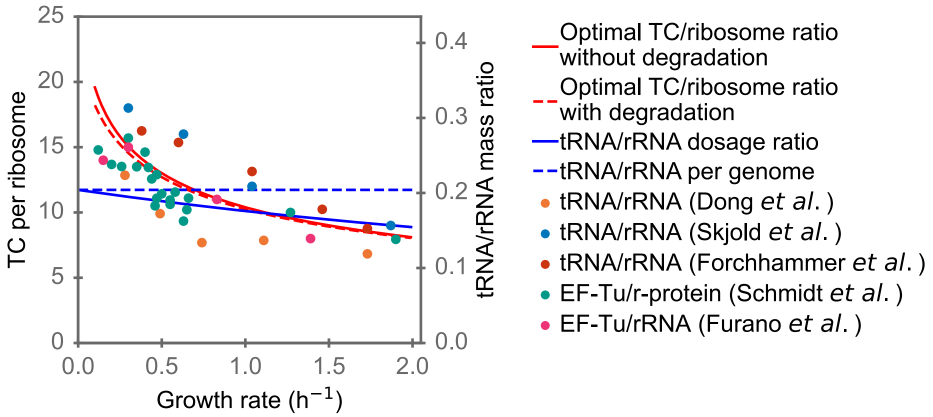

Fig A. The effect of protein degradation on the RNA growth law in *E. coli*. The solid red curve shows the RNA composition growth law as given by equation (1), which ignores protein degradation, whereas the dashed red curve shows the RNA composition growth law with protein degradation included. Protein degradation rate typically ranges from 0.02 h^{-1} to 0.04 h^{-1} [1,2] (see estimation below) and is higher at low growth rates than at high growth rates [1–3]. We here assumed a constant protein degradation rate (k_{deg} = 0.04 h^{-1}); its inclusion is equivalent to moving the optimal TC/ribosome ratio 0.04 h^{-1} to the left. As the degradation rate is relatively small compared to the maximal growth rate of *E. coli* [1,2], protein degradation affects the optimal TC/ribosome ratio only at very low growth rates, where the degradation rate becomes comparable to the growth rate.

Estimation of the protein degradation rate: Publications [1,2] report the fraction of degraded protein (f_{deg}) after time t. The protein degradation function can be written as $1 - f_{\text{deg}} = e^{-k_{\text{deg}} \cdot t}$. With this equation, k_{deg} was estimated to range from 0.005 h^{-1} to 0.04 h^{-1} according to the data reported in [2] and to range from 0.025 h^{-1} to 0.03 h^{-1} according to the data reported in [1]. To be conservative, we here chose k_{deg} = 0.04 h^{-1}, the largest reported degradation rate.

c) *Another minor comment for Fig. 2ef: I wonder if results would be better shown in double-log scale (including the lack of correlation between tRNA/chromosome and mu_max for fast growing cells). The authors can easily provide a supplementary figure.*

Action: As suggested, we now show the plots that were Fig 2E and 2F on double-log scales; these are now Fig 3D and 3E.

Reviewer #3

I performed this review with two close collaborators on this topic.

In this study, Hu and Lercher describe a previously overlooked growth law linking the ratio of tRNA to rRNA to growth rate. After deriving the law (Eq. 1) from an optimality hypothesis and based on a previous (but unpublished) result (ref. 9), they show that the law holds using data from E. coli (Fig 1a) and across microbes (Fig 1b). Then they use the Cooper-Helmstetter model to show that a prediction of tRNA to rRNA ratio based on gene dosage reproduces the optimal prediction (quantitatively at fast growth) across conditions and species (Fig 1b and Fig 2).

The reasoning is convincing, the results appear to be quite solid and our overall impression of the study is positive, although the text is rather condensed and it could be clearer. Definitely this study fits the standards of the journal and it would make an interesting read for anyone interested in cell growth and its impact across genomes.

We propose below some revision points that in our opinion could make the study stronger and easier to read

***Response**: We thank the reviewers for this positive evaluation.*

Major Comments / Questions

1. *Should this result justify the introduction of a concept of "RNA sectors" and their partitioning?*

Response: We thank the reviewers for the suggestion. However, as shown in the response to the next comment, the RNA growth law only influences the prediction of growth rate at low growth rates, and even there only has a minor effect when comparing to the assumption of a constant tRNA/ribosome ratio (4.4% difference at μ = 0.2 h^{-1}). Accordingly, while tRNA and rRNA are expressed near the optimal ratio, the introduction of an RNA growth law will only have very minor effect on the prediction of the cellular growth rate. We thus did not attempt to introduce the RNA growth law into cell growth models.

Action: We now discuss this in the main text (in subsection "Impact of the RNA growth law on cell growth and genome organization", line 264) and in supporting information (Text D in S1 File).

2. *The authors mention that other studies on growth laws assumed the tRNA to rRNA ratio to be constant. It would be really interesting to know whether the growth law they find has an impact on other growth laws that were derived based on the constancy assumption, and whether the discrepancy can be observed in some data (for example data from Dai et al 2016).*

Response: Briefly, our assumption of an optimal tRNA/rRNA ratio and the assumption of a constant ratio lead to very similar growth rate predictions. However, as translation is a very expensive process, the small difference is highly relevant in evolution.

Assuming a constant protein concentration across conditions in *E. coli*, we first calculated the optimal concentrations of ribosome and TC based on equation (9) and (10), respectively. The

summed density of ribosome and TC was calculated by providing the molecular weights of ribosome and TC.

We then calculated the concentration of ribosome and TC under the constant assumption with $\mu \cdot [P] = k_{eff} \cdot [R]$, where μ is growth rate, $[P]$ is protein concentration, $[R]$ is ribosome concentration, and k_{eff} is the effective turnover rate of the ribosome. The constant assumption assumes the rRNA:tRNA mass ratio is 86:14 (Bremer and Dennis 2008, EcoSal Plus 3: 765); and this results in the constant TC:ribosome molar ratio 9.6:1. Further, $k_{eff} = k_{cat} \cdot K_M/([TC]+ K_M)$, with k_{cat} (22 s^{-1}) as the ribosome turnover rate, $[TC]$ as the concentration of TC and $[TC]$ = 9.6 [R], and K_M (122 µM) as the Michaelis constant (see Methods). The ribosome concentration [R] can be obtained by solving $\mu \cdot [P] = k_{eff} \cdot [R]$; the summed density of ribosome and TC can be readily calculated with solved $[R]$ and $[TC]$.

By comparing the cytosol density of the optimal TC/ribosome ratio and the constant TC/ribosome ratio at the same growth rate (protein synthesis rate), we found that the major difference is at low to moderate growth rates, whereas the cytosol densities of the two assumptions are very similar at high growth rate (see Fig J in S1 file, copied below).

Action: We now discuss this in the main text (in subsection "Impact of the RNA growth law on cell growth and genome organization", line 264) and the supporting information (Text D in S1 File).

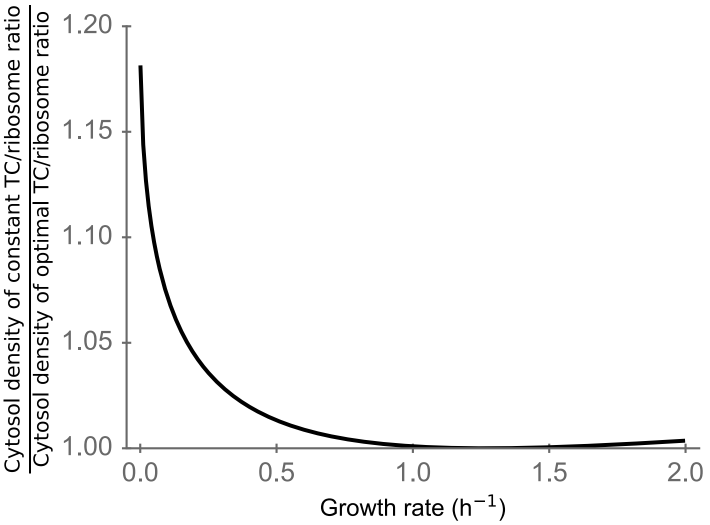

Fig J. Cost difference (in terms of dry mass density) between the optimal TC/ribosome expression ratio and a constant TC/ribosome expression ratio in *E. coli* as a function of growth rate.

3. *The authors are basically looking at how tRNAs are repartitioned among available ribosomes (tRNA/rRNA). What will happen if we compare tRNA per *translating* ribosome? Should it be a constant?*

Response: Our model assumes that all active ribosomes (i.e., all that are not de-activated, which *E. coli* does for a fraction of ribosomes at low growth rates (Dai *et al.* 2016)) are bound to mRNA and are translating, i.e., in the process of elongating a peptide chain. It should be noted that "tRNA" in the measured tRNA/ribosome ratio is the total tRNA, whereas "tRNA" in our predicted optimal tRNA/ribosome is the "active tRNA", i.e., the tRNA bound in ternary complexes. In our previous

work, we found that there is a substantial fraction of inactive tRNA and ribosome at low growth rates (Hu et al. 2020). This approximation may contribute to the discrepancy between our predictions and data at low growth rates.

Action: We now discuss these issues in the manuscript (line 119).

4. *Some central results rely on previous results by the same group, but the text should be made more self-contained. In particular:*
   a) *Eqs [4] and [5], which give Eq [1] come almost directly from ref. 9. It would make the life of a reader much easier if they were at discussed and motivated a bit more, at the cost of a small overlap with ref 9 (oddly this is only a preprint although quite old)*

Action: We now summarize the derivation of equation (1) in the Methods section (from line 323).

   b) In the current text the hypothesis of optimality is not sufficiently discussed and clarified, both in the results and in the discussion. Again, it is clear to us that this is part of past work, but in our view the extra explanations are required to make this study a bit more self-contained.

Action: We extended the explanation in the revised manuscript (line 75).

5. *A big caveat on the dosage results is that (at least for E. coli), the C period has a strong dependency on temperature (we are modelers ourselves, but we believe it could be a factor of 3 from 37 to 28 degrees), which is a source of variability in the data. This point should be raised by the authors. It can also be addressed in the model, at least of E. coli, looking at the predictions vs temperature and possibly also going back to data from experiments performed at different temperatures.*

Response: Briefly, our model does not work at low temperatures in *E. coli*, possibly because low temperature is perceived as a stress condition by this organism. We found a dataset for the temperature dependence of the C period in *E. coli*, with estimates at 14, 21, 30, and 37 °C in the same medium (Atlung and Hansen, Journal of bacteriology 1999, 181: 5557). The DNA replication rate became slow at low temperature, and multiple replication rounds were observed even at low growth rates. Accordingly, at low temperatures, the *E. coli* tRNA/rRNA dosage ratio becomes almost independent of the growth rate (Figure F in S1 File, copied below); thus, this ratio does not obey the predicted relationship (solid blue line) under temperature stress conditions.

Action: We discuss this as a model limitation in the revised manuscript (line 287) and in Supporting information (Fig F in S1 File).

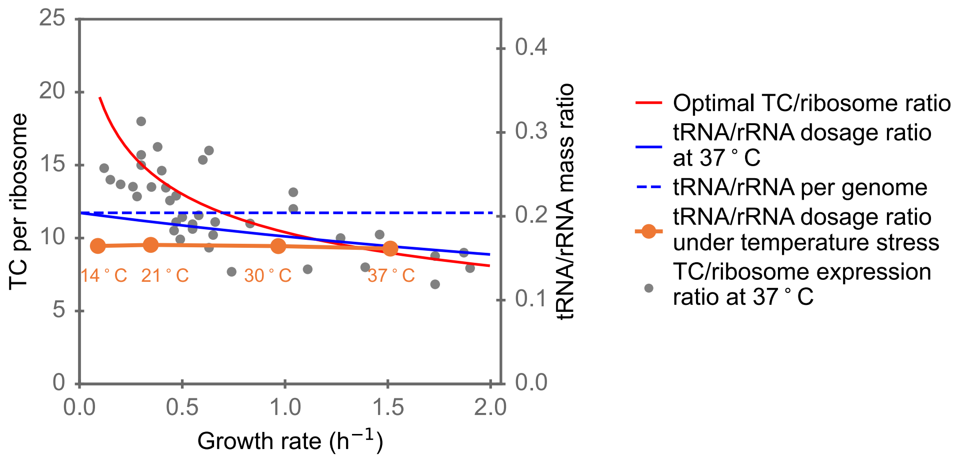

Fig F. The tRNA/rRNA gene dosage ratio is independent of growth rate under temperature stress conditions. The orange dots show experimental measurements at 14 °C, 21 °C, 30 °C, and 37 °C in the same medium [6]. At the low temperatures, the DNA replication rate became slow at low temperatures, and multiple replication rounds were observed even at low growth rates. This effect made the rRNA/tRNA dosage ratio almost independent of growth rate. As we could not find experimental data for the tRNA/Ribosome expression ratio under temperature stress conditions, it is unclear if the tRNA/ribosome expression ratio is still optimal under temperature stress conditions.

More specific comments to improve clarity and communication

6. *Can the authors clarify how *"the observation that the cellular dry mass density is approximately constant across growth conditions"* leads to the hypothesis of a *" minimization of the total mass of all participating molecules "*.*

Response: The naïve idea is the parsimonious usage of dry mass density of a particular cellular process. As the cellular dry mass density is nearly constant across growth conditions, if the dry mass density is occupied by one particular molecule, less will be available for all other molecules. Further, as translation accounts for a large fraction of cellular dry mass. It is likely the dry mass density of summed translational components is minimized at the given protein synthesis rate.

Action: This is now explained (line 75).

7. *Figure 1. It seems the authors should detail how they computed the solid and dashed blue lines. In panel b, model does not seem to do an excellent job, at least looking how the data are presented now, maybe the discrepancies should be discussed.*

Response: The data in panel b (now Fig 2A) were measured by different groups over a large timespan; this could be responsible for discrepancies in the data. Inspired by the reviewer's next comment (see our response there), we extended our analysis of Fig 2A to explore the consistency between the model fits and the data.

We found the data for *Streptococcus pyogenes* to be unsuitable for our analysis. Our model depends on the diffusion-limited binding rate of between TC and ribosome ($k_{\text{on}}^{\text{diff}}$), which is

assumed to be a constant in normal osmotic conditions. However, the tRNA/ribosome ratios for *Streptococcus pyogenes* were measured under different osmotic pressures (Panos *et al.* 1965, Biochem Biophys Res Commun 19: 62). As the osmotic pressure affects the cellular dry mass density and hence the diffusion rate of all cytosolic components, $k_{\mathrm{on}}^{\mathrm{diff}}$ will also change for this species under the studied conditions. We thus excluded this species in the new version of the manuscript.

Action: We explained how the solid and dashed lines in Fig 1B (formerly Fig 1a) are calculated in the text (line 147); for further analyses, see our "Action" in response to the next comment.

8. *Connected: with the data in Fig 1b one could try a data collapse based on Eq [1], to show that the data are compatible with a "universal" law across species. For example one could easily collapse by r, and show that two clusters (plus Synechococcus) appear, then extract a and look at the values (but possibly something much smarter can be done…)*

Response: Motivated by the reviewer's comment (and a related comment from Reviewer 2), we examined equations (1) and (2) further, finding that mathematically identical versions can be formulated with only one species-specific parameter, the ribosomal turnover number k_{cat}. We then estimated these k_{cat} values by fitting the re-formulated equation (1) to the data points in Fig 1B and Fig 2A. In support of our model, we found that the fitted k_{cat} values are statistically significantly correlated with measured ribosome effective turnover number (k_{eff}) values.

More specifically: as the binding between the ribosome and the TC is limited by the diffusion of the TC, K_{m} can be approximated by $K_{\mathrm{m}} \approx k_{\mathrm{cat}}/k_{\mathrm{on}}^{\mathrm{diff}}$, with $k_{\mathrm{on}}^{\mathrm{diff}}$ the diffusion-limited binding rate (Klumpp *et al.* 2003, PNAS 110: 16754). Thus, $k \coloneqq \sqrt{k_{\mathrm{cat}} \cdot K_{\mathrm{m}}} \approx k_{\mathrm{cat}}\sqrt{1/k_{\mathrm{on}}^{\mathrm{diff}}}$. As the cytosolic density of macromolecules (and hence diffusion) is similar across microbes, we can assume that $k_{\mathrm{on}}^{\mathrm{diff}}$ is (approximately) a constant. Equation (1) and (2) only contain the species-specific parameter k_{cat} , and equation 1 can be rewritten as

$$\frac{[TC]}{[R]} = \frac{a \cdot k_{\mathrm{cat}}\sqrt{1/k_{\mathrm{on}}^{\mathrm{diff}}}}{\sqrt{a \cdot \mu \cdot [P]} + k_{\mathrm{cat}}\sqrt{1/k_{\mathrm{on}}^{\mathrm{diff}}}} \qquad \text{(S1)}$$

Reformulating equation (S1), we can get equation (S2), which is equivalent to equation (1) in the main text:

$$\frac{[TC]}{[R]} = \frac{a \cdot k_{\mathrm{cat}}}{\sqrt{a \cdot \mu \cdot [P] \cdot k_{\mathrm{on}}^{\mathrm{diff}}} + k_{\mathrm{cat}}} \qquad \text{(S2)}$$

We used least squares (Levenberg–Marquardt algorithm) to fit the measured data of each species (in Fig 1B and Fig 2A) to equation S1 to estimate k_{cat} for each species. To compare these estimates to independent experimental values, we estimated the effective ribosome turnover number (k_{eff}) by $\mu \cdot [P] = k_{\mathrm{eff}} \cdot [R]$, where μ is the growth rate, $[P]$ is the protein concentration, and $[R]$ is the ribosome concentration (data in S4 Table).

We exclude the species *Streptococcus pyogenes* in this analysis. Its tRNA/ribosome ratio decreases with increasing growth rate, which is consistent with our theory. However, our model depends on

k_{on}^{diff}, which is assumed to be a constant on normal osmotic conditions. The tRNA/ribosome for *Streptococcus pyogenes* were measured under different osmotic pressures (Panos *et al.* 1965, Biochem Biophys Res Commun 19: 62). As the osmotic pressure affects the diffusion rate of all cytosol components, k_{on}^{diff} will also change for this species under the studied conditions, violating our assumptions. We also excluded *Synechococcus elongatus* from the fitting, as the data shows no significant correlation between growth rate and the tRNA/rRNA ratio.

The Pearson correlation between the k_{cat} values fitted according to our theory and k_{eff} estimated from independent experimental data is $r = 0.79$ ($P = 0.06$; Fig 2B, copied below). Since both k_{cat} and k_{eff} estimates rest on (different!) simplifying assumptions, and because the tRNA/ribosome ratios used for fitting equation (S1) were measured with different methods by different groups, we do not expect a perfect correlation; that we still get an r^2 of 62% appears to strongly support our analyses.

Action: We discuss these analyses in the main text (line 178, Fig 2B) and conclude that equation (2) is indeed a universal RNA growth law across species.

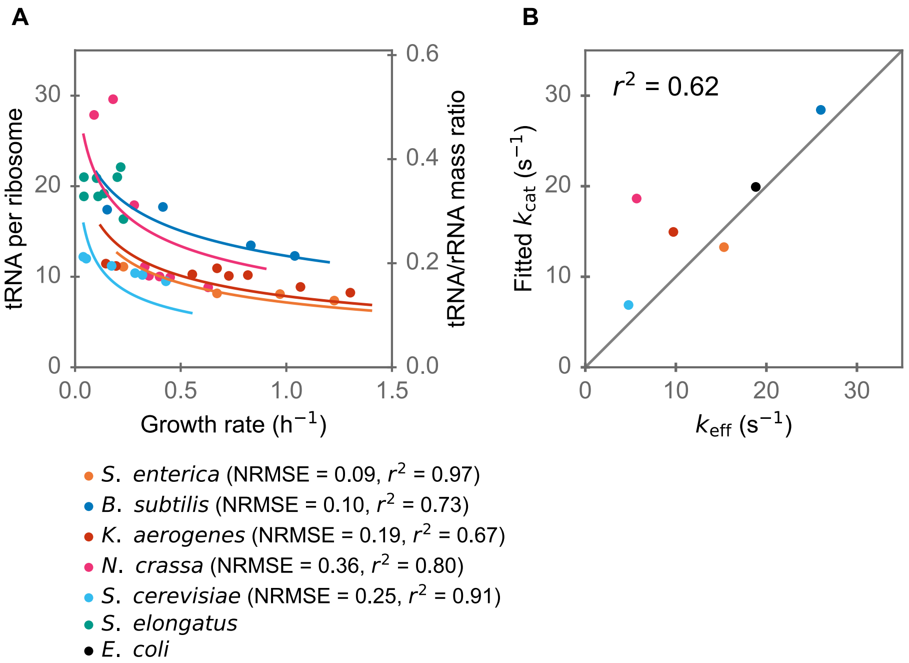

Fig 2. The RNA growth law across species. (A) Experimentally observed tRNA/ribosome expression ratios in different microbes decrease with growth rate, consistent with the predicted optimal tRNA/ribosome expression ratio. For each species except *S. elongatus*, which is a slow-growing species and shows no systematic growth rate dependence, we fitted equation (1) to the data by varying the single adjustable parameter k_{cat} (colored lines). Note that the y-axis on the right-hand side is based on the tRNA/rRNA mass ratio for bacteria. For eukaryotic microbes, the tRNA/rRNA mass ratio should be scaled by a factor of 0.84 according to equation (15). (B) Comparison of fitted k_{cat} and effective ribosome turnover number k_{eff}.

9. *Line 103. The discussion about gene dosage appears to start out of the blue. It is however linked with what said before. Can the authors improve the transition for the reader?*

Action: We improved the transition in the new version (line 131).

10. *Line 110. *"Here and below, for each genome, we summarize the multiple tRNA genes by averaging over their positions; we do the same for the rRNA genes."* I can see how that simplifies the analysis, but the risk is to wash out important gene-dependent regulation, codon bias, etc etc. Maybe for a future work?*

Response: It is not obvious how best to combine the individual positions into a simple metric for each genome. When calculating gene dosage, however, we did so while considering the positions of individual genes (Fig 3D and equation (21)).

We tested for an influence of codon frequencies on the positions of individual tRNA genes: there is no correlation between the position of a tRNA and its codon frequency in most species. For a specific tRNA in a given genome, we identified the anticodons by tRNAscan-SE 2.0 and used the wobble paring rule to find the cognate codon(s) (codons that can be translated by this tRNA). Then, the frequency of a tRNA's cognate codon(s) was calculated as the summed frequency of all its cognate codon(s). We then performed correlation tests between tRNA positions and the associated cognate codon frequencies in a given species.

As expected, the <u>number</u> of tRNA genes per tRNA species showed a positive correlation with the genomic frequencies of the corresponding codons for practically all fast-growing species ($p < 0.05$ in 45 out of 48 species), and also for 37 out of 122 slow-growing species (panel 2 of Fig H in S1 file, copied below). For most fast-growing species, we also found a negative correlation between the <u>positions</u> of tRNA genes and the corresponding codon frequencies (35 out of 48); for 12 of these, the correlation was statistically significant ($p<0.05$). Overall, we found statistically significant negative correlations ($p < 0.05$) between tRNA genomic positions and the frequencies of cognate codon(s) in only 15 out of the 170 species analyzed (panel 1).

However, it is not obvious to us which pattern to expect: the optimal dosage of all tRNAs should increase in sync with growth rate, so that the optimal genomic position would in fact be identical for codons with high and low frequencies. However, natural selection may act more strongly on those tRNA genes that encode more abundant tRNAs, potentially explaining the observation of a negative correlation in most fast-growing species.

Action: The positions of individual tRNA and rRNA genes in *E. coli* are shown in Fig C in S1 File (copied below), with the mean genomic position 0.20 ± 0.17 (mean ± standard deviation) for rRNA genes and 0.45 ± 0.27 for tRNA genes. The positions for the other species are provided in S3 Table. We briefly report the analyses concerning codon frequencies in line 298 and more extensively in Text B in S1 File.

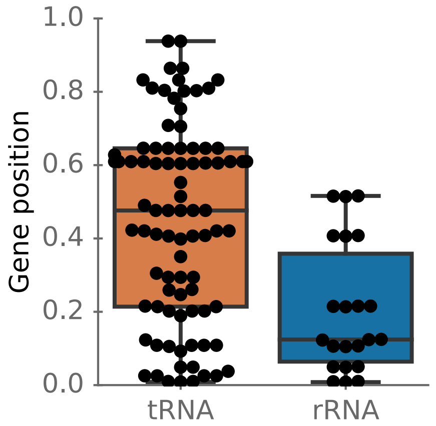

Fig C. Gene positions of rRNA and tRNA genes in E. coli. rRNA genes are located closer to oriC than tRNA genes in *E. coli*, with genomic position 0.20 ± 0.17 (mean ± standard deviation) for rRNA genes and 0.45 ± 0.27 for tRNA genes.

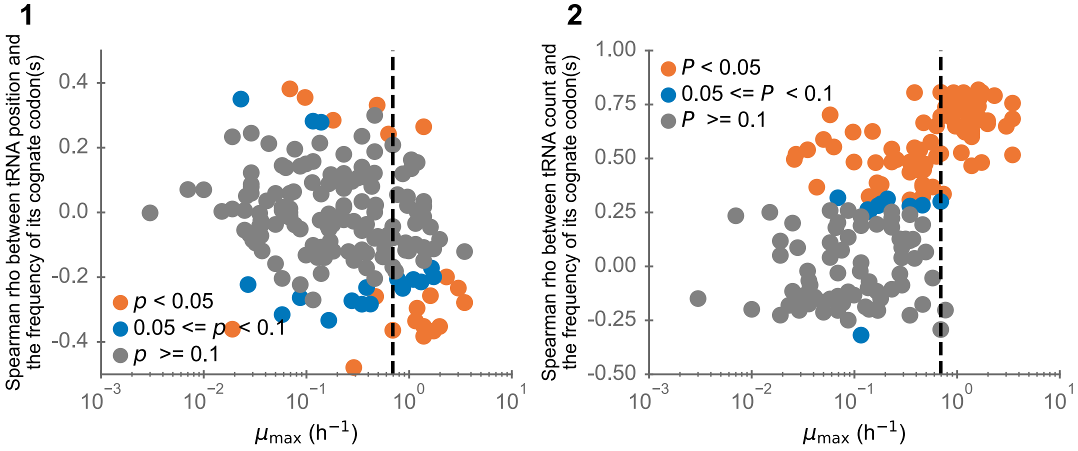

Fig H. Copy numbers of tRNA genes but not their positions are correlated with codon frequency. Each datapoint represents Spearman's rank correlation coefficient ρ for a single genome. Point color indicates statistical significance. (**panel 1**) Correlation between tRNA position and cognate codon frequency. (**panel 2**) Correlation between tRNA genomic copy number and cognate codon frequency.

11. *In Eq(16) we did not understand why there is n for the ribosomes but not the equivalent for tRNAs.*

Response: One ribosome contains three rRNA genes (5S, 16S, and 23S rRNA), thus the number of rRNA genes divided by 3 is the number of ribosome copies.

Action: This fact is now made explicit in the manuscript (line 393 in Methods).

12. *Line ~135. *"Here, the tRNA/rRNA dosage ratio at higher growth rates (1h/0 ≤ μ ≤ 2h/0) is very close to the optimal prediction, which corresponds to about 9 tRNAs per ribosome. "* I might have lost it, but from where?*

Response: This sentence describes the red curve in Fig 1B (originally Fig 1a) for growth rates from ~1 to ~2 h^{-1}.

Action: We clarified the text (line 155).

13. *Just after, there is something about the lines in Fig.1, which are actually not explained.*

Action: In the revised version, we explain all data and prediction lines in Fig 1; we explained how the solid and dashed lines in Fig 1B (formerly Fig 1a) are calculated following line 147.

Additional Minor Comments

14. *A sketch or cartoon of the Michaelis-Menten model used to derive Eq [1] could be useful to help the reader.*

Response: We agree that such a cartoon would be helpful; however, we feel that another type of schematic diagram will be more useful to the reader.

Action: We now explain the derivation of equation (1) in Methods, re-drew Fig 1, and include a schematic diagram for the optimization in Fig 1.

15. *In our view Fig 1a is arguably the most important of this study. It could be given more graphical emphasis / graphical explanation.*

Action: Motivated by the reviewers' comment, we rearranged Fig 1. We added a new panel (Fig 1A) that describes how we derive the optimal TC/ribosome ratio. We moved the original Fig 2A to Fig. 1 (panel C) to highlight the growth rate-dependent tRNA/rRNA dosage ratio. Fig 1B then shows how these two agree with each other and with data from *E. coli*.

16. *The authors use TC and not tRNA data. This includes elongation factor Tu (EF-Tu), GTP, and _charged_ tRNA (so it depends on aa). Is this a problem?*

Response: According to our assumptions, it is the TC concentration for which we can predict optimality. In the coarse-grained model for peptide elongation, the ribosome acts as the enzyme and the TC acts as the substrate. Both ribosome and TC consist of protein and RNA; to assess the resource efficiency of elongation, we must therefore consider the costs of protein and RNA. In previous work, we found that the molecular weight of ribosome and TC are good proxies for their costs to the cell (Hu 2020) (e.g., better than using the protein content, see also Fig B in S1 File).

Action: These topics are now discussed in the manuscript (line 127).

17. *Is it possible to make a back of the envelop estimate of μ_{max} from the position of rRNA and tRNA genes?*

Response: Unfortunately, we cannot estimate μ_{max} with any reasonable accuracy from the positions of tRNA and rRNA genes; the highest correlation coefficient is only ~ 0.6 (Spearman ρ = 0.59 and Pearson r = 0.56 for the correlation between rRNA position and log(μ_{max})), i.e., the position explains only r^2 = 31% of the variation in μ_{max}.

Action: none.

18. *Fig.2 The choice of μ_{max} to distinguish slow fast is in principle arbitrary, but from (for instance) tRNA position it is evident. Is there something behind that?*

Response: The threshold (1 h) is roughly the sum of the replication time (C ≈ 40 min) and division time (D ≈ 20 min) in *E. coli*. Species with minimal doubling times lower than this threshold are very likely to have multiple replication rounds per cell cycle; accordingly, replication-associate gene dosage effects are expected to be strongest in these fast-growing species.

Action: We now explain the rationale for our choice of threshold in the text (line 195).

19. *The result on gene dosage appears across Fig 1b and 2. Maybe a more optimal figure order can be attempted.*

Action: We rearranged the figures, as suggested.
