# Supplementary material for: An optimal growth law for RNA composition and its partial implementation through ribosomal and tRNA gene locations in bacterial genomes
Source: PLoS Genet. 2021 Nov 29;17(11):e1009939. doi: 10.1371/journal.pgen.1009939 (PMC8659690; doi:10.1371/journal.pgen.1009939)
Supplement: Attachment — Submitted filename: Hu_Response2Reviewers2.pdf [file pgen.1009939.s008.pdf]

Reviewer #2

By addressing my comments and the comments from the other reviewers, the Authors have greatly improved the manuscript. I think that the revised manuscript adheres to the high standards of quality and broad relevance of PLOS Genetics, and I am happy to recommend its publication.

Response: We thank the Reviewer for this positive assessment.

Reviewer #3

I am happy with the authors' replies and revisions.

Response: We thank the Reviewer for this positive assessment.

Some minor comments that the authors might optionally consider for a brief discussion

1. *In this recent study, the authors derive a tRNA growth law (Eqs 6-9), which might be interesting to compare with this study*
   PMID: 34389683

Response: Roy *et al.* (PMID: 34389683) derived "growth laws" for bacteria from a unifying autocatalytic network framework under balanced exponential growth (steady state) conditions. The growth laws they propose – including the one for tRNA – emerge directly from the steady state of the autocatalytic cycles; they are independent of any optimal or non-optimal resource allocation beyond the steady state requirement. Moreover, the tRNA growth law contains many parameters that might be very difficult to measure.

In contrast, our growth law was derived from optimal resource allocation, resulting from a minimization of the total cost of ribosomes and ternary complexes at a given protein synthesis rate. Our growth law only contains a single species-specific parameter, the turnover number of ribosome k_{cat}. The resulting, optimal concentrations of tRNA and ribosome, as well as the tRNA/ribosome ratio can be easily calculated at a given growth rate.

We thus feel that the tRNA growth law in *Roy et al.* is conceptually too different from ours to allow a meaningful comparison in our manuscript.

Action: none.

2. *One additional caveat that may be mentioned is the physiological dependency of the C period on growth rate*
   PMID: 32424336
   PMID: 12686642

Response: As pointed out by the Reviewer, the C period is indeed growth rate-dependent. In *E. coli*, it is almost constant at growth rates above 0.7 h^{-1} but increases with decreasing growth rate below

0.7 h^{-1} (Panel 1 of the new supporting **Fig G**, copied below; data from the two papers pointed out by the Reviewer, PMID 32424336 and 12686642). However, as shown in Panel 2, when we calculate the tRNA/rRNA dosage ratio using the experimentally observed rate-dependent C period, the results are very similar to those calculated under the assumption of a constant C period and DNA replication rate (k_{rep} = 1000 s^{-1}) (calculated by equation (21)). Thus, setting k_{rep} = 1000 bp s^{-1} appears to represent an acceptable approximation for the tRNA/rRNA dosage ratio calculation.

Action: We now discuss the effect of a growth rate-dependent C period on the tRNA/rRNA dosage ratio in the supporting information (Text A with **Fig G** (copied below), referred to in line 140 in the revised manuscript).

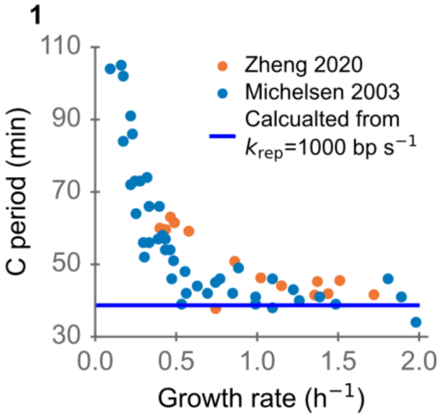

Fig G. The effect of a growth rate-dependent C period on the tRNA/rRNA dosage ratio in *E. coli*. (**panel 1**) Comparison of the C period calculated from constant k_{rep} = 1000 bp s^{-1} (solid blue line) and experimental estimates of the C period (orange and blue dots). (**panel 2**) The growth rate-dependent C period has only a minor effect on the predicted tRNA/rRNA dosage ratio. The blue line shows the dosage ratios calculated under the assumption of a constant C period. The orange and blue dots show dosage ratios calculated using the respective experimental C period values shown in panel 1.

3. *There are two other attempts (from the same group) of cross species growth law that the authors might want to compare with their approach*
   PMID: 27046336
   PMID: 22203990

Response: We thank the reviewer for referring us to these two interesting papers. The methodology used by the authors is very different from our optimization approach. In the second paper, Kempes *et al.* (PMID: 22203990: "Growth, metabolic partitioning, and the size of microorganisms", PNAS 2012) estimate the relationship between species-specific growth rate and the body size with two empirical functions (the Pirt model and the total metabolic rate function). Kempes *et al.* then build on this relationship to derive empirical relationships for the volume occupied by different types of macromolecules (DNA, ribosome, protein, tRNA, mRNA, and cell membrane) across bacterial species (PMID: 27046336: "Evolutionary tradeoffs in cellular composition across diverse bacteria", ISME J. 2016). For example, based on the assumption that the molar concentrations of tRNA, mRNA, and

ribosome are identical (1:1:1), they describe both DNA volume and protein volume as empirical functions of the cell volume.

Action: We now cite these papers in the introduction (line 47, References 12 and 13).

4. *Concerning the role of degradation (asked by another referee), there is a preprint* *https://doi.org/10.1101/2021.03.25.436692*

Response: Our estimate of the protein degradation rate is consistent with this paper (about 0.04 h^{-1} at low growth rates), as the same original data (Pine 1973) was used in both this preprint and our manuscript.

Action: We now cite this preprint in the revised manuscript (line 117, Ref. 54).
